# Polymers and Plastics Modified Electrodes for Biosensors: A Review

**DOI:** 10.3390/molecules25102446

**Published:** 2020-05-24

**Authors:** Sonia Lanzalaco, Brenda G. Molina

**Affiliations:** 1 Departament d’Enginyeria Química, EEBE, Universitat Politècnica de Catalunya, C/ d’Eduard Maristany, 10-14, Building I, E-08019 Barcelona, Spain; 2 Barcelona Research Center in Multiscale Science and Engineering, Universitat Politècnica de Catalunya, Campus Diagonal Besòs (EEBE), C/ d’Eduard Maristany 10-14, Edifici IS, 08019 Barcelona, Spain

**Keywords:** conducting polymers, modified biopolymers, modified bioplastics, recyclable plastics, flexible electrochemical biosensors

## Abstract

Polymer materials offer several advantages as supports of biosensing platforms in terms of flexibility, weight, conformability, portability, cost, disposability and scope for integration. The present study reviews the field of electrochemical biosensors fabricated on modified plastics and polymers, focusing the attention, in the first part, on modified conducting polymers to improve sensitivity, selectivity, biocompatibility and mechanical properties, whereas the second part is dedicated to modified “environmentally friendly” polymers to improve the electrical properties. These ecofriendly polymers are divided into three main classes: bioplastics made from natural sources, biodegradable plastics made from traditional petrochemicals and eco/recycled plastics, which are made from recycled plastic materials rather than from raw petrochemicals. Finally, flexible and wearable lab-on-a-chip (LOC) biosensing devices, based on plastic supports, are also discussed. This review is timely due to the significant advances achieved over the last few years in the area of electrochemical biosensors based on modified polymers and aims to direct the readers to emerging trends in this field.

## 1. Introduction

Owing to their potential usefulness in personal healthcare and disease diagnosis, medical biosensors widely attract the attention of the research community [1,2]. These devices can diagnose a wealth of diseases and health conditions, such as diabetes, cardiovascular issues, infectious diseases and cancer [3,4,5]. An interesting report of IDTechEx, recently published, predicts that the market for biomedical diagnostics is expected to grow steadily, reaching $43 billion by 2029 [6], due to a growing and aging global population increasing health expenditure and more prevalent lifestyle such as obesity, cancer and cardiovascular diseases. This report presents a list of new technologies and devices which are likely to be highly disruptive to the in vitro diagnostics market, including microfluidic lab-on-a-chip, lateral flow assays, electrochemical test strips, molecular diagnostics and DNA sequencing [6].

Typical medical biosensors rely on the coupling of (*i*) a biological recognition element (enzyme, antibody, oligonucleotide, aptamer) interacting selectively with the target biomolecules, and, (*ii*) a suitable transducer that monitors the degree of this interaction, providing qualitative and quantitative information [1]. The versatility of these devices can be enhanced through layered construction by combining two or more materials of different properties. In this review we focus our attention on biosensors which employ electrochemical circuits to detect biomolecules. The electrochemical modes of detection (amperometric, voltammetric, field effect transistor (FET)-based potentiometric or impedimetric) are perfectly suited and enable high selectivity and sensitivity in biosensing devices [7,8,9,10,11]. In contrast to other techniques such as microdialysis and nuclear magnetic resonance (NMR) spectroscopy, electrochemical sensors can rapidly and precisely measure extracellular low analyte concentrations, if used, for example, within the tissue in near real time. Amperometric electrochemical sensors are based on the application of an electric potential and the measurement of an electrical output signal (the measured current) that is proportional to the analyte concentration. One of the most important class is represented by the enzyme-based, amperometric, electrochemical biosensing able to highly selective and sensitive response in a complex environment [12]. These chemical sensors are called biosensors because enzymes with their very specific interaction with a substrate are immobilized as biological recognition elements onto electrodes. The most widely studied in vivo sensors are continuous glucose monitoring systems aimed at patients with metabolic disorders (e.g., diabetes) [13,14]. The other main driving force behind the development of sensors is the neurosciences. Many sensors have been developed for short-term brain application in animal models, mostly for neurotransmitters (e.g., glutamate or choline) and energy metabolites (e.g., lactate) [15,16,17,18,19].

Previous reviews have widely discussed general fabrication aspects and other technical issues associated with biosensors [20,21,22,23]. Different materials can be used in the fabrication of these devices, but very interestingly, in the last decades, a great attention has been addressed to polymers and study based sensors [24,25]. Polymer materials have several intriguing advantages when considered as support platforms for biosensors: they are lightweight, ultra-conformable (bendable, stretchable, foldable), portable, disposable and inexpensive. Furthermore, they offer extended scope for a high degree of functional integration and, thus, can accommodate additional functionalities (i.e., wireless transmission modules, control and data acquisition instrumentation, in-built power units, etc.) and have advantages over metallic and ceramic materials, such as mild synthetic conditions, scalable and large-area processing, low operating temperature and biocompatibility [26].

Finally, polymers could be easily modified in order to improve key requirements, necessary to expand their applications. The most important parameters for sensing performance are sensitivity, response/recovery time and reversibility/reproducibility of response, which are strongly dependent on the chemical structure and size of the polymers. From both an engineering point of view and a sensor development point of view, we consider that is very useful to make a review related to the emerging field of biomedical sensors based on electrochemical detection and produced by using polymeric material subjected to surface and structural modification.

**Figure 1:**
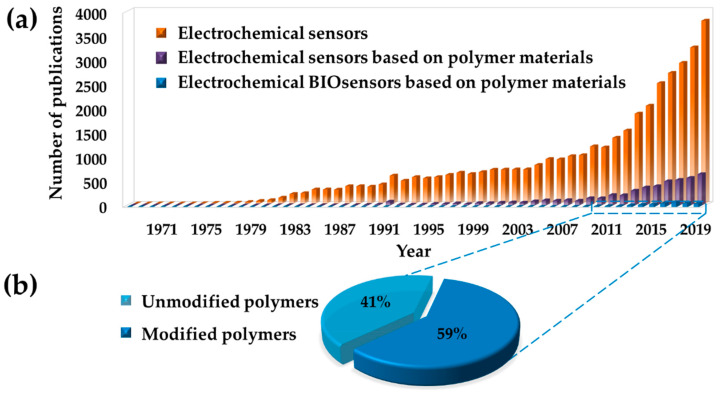
(**a**) Comparison between the evolution of electrochemical-based sensors published research papers per year in the last decade, based on polymer materials and employed in the biomedical field and (**b**) percentage of biosensors based on electrochemical detection composed made of unmodified and modified polymers. Source: Web of Science (WOS).

This review focuses on significant works over the last 10 years that could potentially determine future trends in the area of biosensors. Figure 1a presents the evolution of the number of papers published, showing an increased attention of the research community in the last 10 years (works published in 2020 have been considered and discussed in the review, but not visualized in Figure 1a because the current number does not represent the entire year). The research is related to sensors based on electrochemical detection and compare them with those produced with polymer material and employed in the biomedical field. The last decade was selected taking into account the huge amount of works encountered and to chiefly update the advances of the topic under study. From this research study, it is possible to realize that over 22,860 papers were published on electrochemical based sensors from 2010, which 3855 (~17%) of them are related to devices composed by polymers. Even the range of time explored is quite short, the number of scientific investigations found is elevate and an exponential growth has been detected, envisaging for continuous growing in the future. Among them, the devices composed by polymers represent the ~14% (536 works found), almost equally divided into modified and not modified polymers, as reported in Figure 1b.

Selected papers and corresponding important parameters as polymer materials employed, type of modification and biomolecules detected, are listed in Table 1.

Prompted by the noteworthy and growing interest registered in the last decade and by the need to explore the potential utilization of modified polymers as electrochemical biosensors, this review compiles and discusses selected highlighted papers of this period. Previous reviews [27,28,29,30,31] have focused the attention on the modification modes, molecules detected. Herein the authors report a review centered on the polymers and plastics employed in the more important sensing system used in the biomedical field, with the aim to direct the readers from sophisticated conducting polymers to emerging trends represented by cheap "environmentally friendly" recyclable plastics as well as bioplastics and biodegradable plastics, modified to be converted into conducting materials.

Therefore, the first part discusses sensors employing electrochemical methods for the detection (electro-BIOsensors) based on conducting polymers, where the modification is carried out to improve the sensing mechanisms, altering both the surface and the entire structure of the polymer material (surface and structural modification).The second part is focused on the description of non-conducting polymers surface modified by means of immobilization of agents responsible for sensing. Finally, a brief discussion about outstanding prototype of biosensors based on electrochemical detection and composed by common plastics is reported.

## 2. Electro-BIOsensors Based on Modified Conducting Polymers

This section will expose selected works in which intrinsically conducting polymers (ICPs) were modified to improve their properties with the aim to maximize the performance as electro-BIOsensors.

The unique structure of ICPs is the responsible of the electrical conductivity that resemble metals, of the low ionization potentials and of the high electron affinity. Their π-orbital system allows electrons mobility through the polymer chain, either by n-type doping (reduction), where an electron is introduced or by the opposite mechanism *p-*type doping (oxidation), where an electron is removed from the valence band creating a “hole charge carrier” [51]. The electron mobility in ICPs, permit a direct route of electron-transfer between the environment and the electrodes surface, acting as an electron promoter and avoiding the need of an electronic mediator [52,53,54]. Therefore, ICPs are highly sensitive to oxidation/reduction reactions thus, in the presence of a redox analytes, their electronic structure manifest changes that activate a sensing mechanism detected by electrochemical methods [53,55], such as chronoamperometry (CA), cyclic voltammetry (CV), differential pulse voltammetry (DPV), electrochemical impedance spectroscopy (EIS), etc.

Among ICPs, those that were employed as materials for sensing devices are polypyrrole (PPy), polyaniline (PAni) and poly(3,4-ethylenedioxythiophene) (PEDOT). Their biocompatibility, i.e., ability to interact with biologic systems without any adverse response and the possibility to be tailored with inorganic and/or organic elements, such as metals, metal oxide nanoparticles, graphene, graphene oxide, carbon nanotubes, biotargets and others polymers, make them ideal biomaterials for several biomolecules recognition [52,55]. including dopamine (DA), serotonin and acetylcholine neurotransmitters, nicotinamide adenine dinucleotide (NADH), glucose, drugs, flavonoids, bioproducts, biomarkers, among others.

### 2.1. ICPs Modified with Metals

Metal and metal oxide nanoparticles offer unique characteristics that can be used to modify polymers and develop high-performance hybrids. Particularly, metal nanoparticles (NPs) show exceptional optoelectrical properties, fast kinetics and easy absorption, behaviors associated to their high ratio between surface area and volume [56,57,58]. However, NPs have the tendency to aggregate, reducing their surface area to volume ratio and, therefore, their effectiveness [58]. In order to overcome this limitation, polymeric materials have been used as supporting matrix leading to a new class of polymer/metal hybrids which exhibit benefits that cannot be obtained by the materials separately.

Metal NPs in electrochemical (bio)sensors have been used to modify conducting polymers, employing structural or surface methods that amplify ICP-sensitivity toward a specific analyte. Poletti Papi and coworkers, merged silver nanoparticles (AgNPs) and PPy through a reversed microemulsion [32]. The structural changes in the conducting matrix ascribed to the metal NPs incorporation, allowed to successfully use this hybrid for a simple and direct electrochemical determination of glucose, reporting a limit of detection (LOD) of 3.6 μM (signal-to-noise ratio of 3), which permitted an accuracy of 99% to 105% in studies with human saliva samples, proposing a new tool for the treatment of diabetes through a glucose levels control.

**Figure 2:**
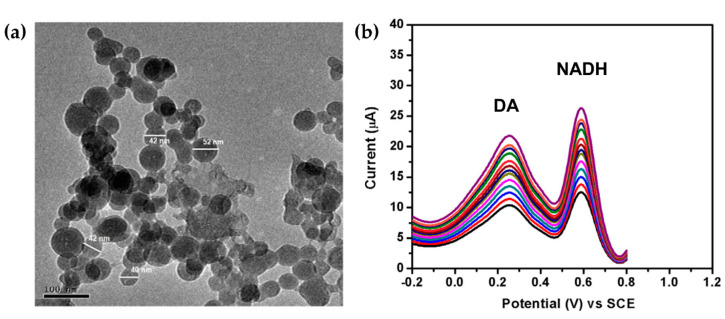
(**a**) TEM micrographs of poly(o-anisidine) (POA)-silver nanoparticles (AgNPs) hybrid (ratio 3:1); (**b**) differential pulse voltammograms of POA-AgNPs in 0.1-M PBS (pH 7.0) containing different concentrations of dopamine (DA) and nicotinamide adenine dinucleotide NADH (from inner to outer). Adapted with permission from reference [59]. Copyright © 2017 Elsevier B.V.

Sangamithirai et al. reported a different method for the structural reinforcement of an ICP matrix, by means of an in situ chemical oxidative polymerization of o-anisidine monomer in the presence of silver nanoparticles [59]. Poly(o-anisidine) (POA), which is a PAni derivative, modified with AgNPs exhibited good electrocatalytic activity due the synergistic effects of both materials. The POA-AgNPs hybrid was able to distinguish between nicotinamide adenine dinucleotide (NADH) and 3,4-dihydroxyphenethylamine (DA) with a LOD = 6.0 nM and 52 nM for NADH and DA, respectively. The precise recognitions of DA neurotransmitter, known to be responsible of several neurological diseases [60] and NADH, metabolic coenzyme involved in cellular energy production [61], plays an important role for the early diagnosis of diseases, for this reason is essential their detection in water samples, human urine or pharmaceutical injections as was proposed by the authors. Figure 2a shows the AgNPs dispersed in POA matrix, while, Figure 2b demonstrates the simultaneously detection of NADH and DA by the hybrid.

Gold nanoparticles (AuNPs), represent another metallic nanomaterial of great scientific interest due to their high catalytic activity and stability [62,63,64]. In fact, it has been reported that AuNPs exhibit a relevant stability for non-enzymatic oxidation of glucose [65,66,67]. Ansari et al. modified poly(aniline blue) (PAB), using it as surface for the seed-mediated growth of AuNPs [33]. As was expected, PAB/AuNPs displayed a good sensitivity detection of glucose (LOD = 0.4 µΜ), the results were associated to the improvement of electron transport properties induced by the synergistic effect of AuNPs and PAB.

**Figure 3:**
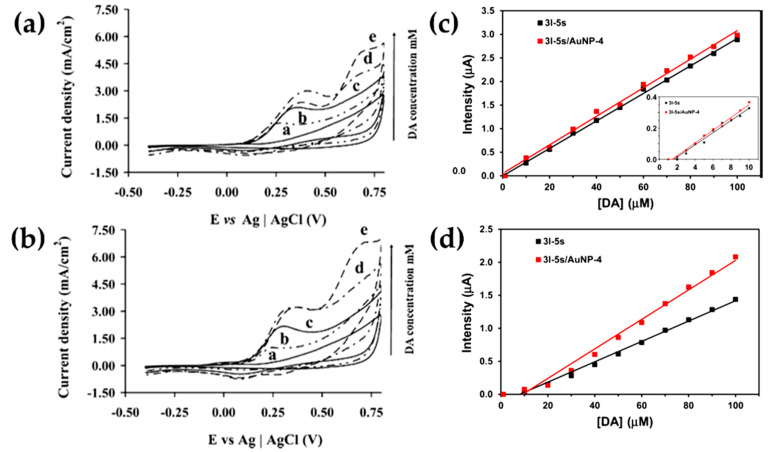
Cyclic voltammograms for the oxidation of (**a**) poly(N-methylpyrrole) (PNMPy)- and (**b**) PNMPy/ gold nanoparticles (AuNPs)-modified carbon electrodes (GCEs) in the absence and presence of different dopamine (DA) concentrations (from 1 to 10 mM). Scan rate: 100 mV/s; initial and final potential: −0.40 V; reversal potential: +0.80 V. For each graphic, labels a-e refer to DA concentrations of 0, 1, 3, 6 and 10 mM, respectively; (**c**) calibration curve for DA concentrations ranging from 1 to 100 μM (inset: from 1 to 10 μM) in 0.1-M PBS and (**d**) calibration curve for DA concentrations ranging from 1 to 100 μM in 0.1-M PBS with 200-μM ascorbic acid and 100-μM uric acid, acting as interferents at), poly(3,4-ethylenedioxythiophene) (PEDOT)/PNMPy/PEDOT (3l-5s) and PEDOT/PNMPy/PEDOT/AuNPs (3l-5s/AuNP-4) electrodes. (a,b) Adapted with permission from reference [68], Copyright © 2011 American Chemical Society and (c,d) from reference [69], Copyright © 2014 American Chemical Society.

Fabregat and coworkers studied the sensing abilities of two PPy derivatives, also modified superficially with AuNPs [68]. Poly[N-(2-cyanoethyl)pyrrole] (PNCPy) and poly(N-methylpyrrole) (PNMPy) were electropolymerized on a glassy carbon electrode (GCE) and coated with a drop of AuNPs colloidal solution. Their results indicated that AuNPs enhance the electronic transference and the charge migration processes of the DA oxidation, although it was only a slight improvement because of the powerful sensing abilities showed by both conducting polymers without NPs. This behavior can be perceived comparing Figure 3a,b, which present cyclic voltammograms of PNMPy without (Figure 3a) and with (Figure 3b) gold nanoparticles, at different concentrations of DA. Following the same research line, the same authors reported the combination of two different conducting polymers and gold nanoparticles [69]. A three-layered sensor was obtained from an electrochemical polymerization, layer-by-layer, of PEDOT and PNMPy, followed by AuNPs colloidal solution dropping onto the external layer of the film. Similar to their previous results, the sensitivity of PEDOT/PNMPy/PEDOT film increased from 5.3 to 6.1 µA/mM DA after the coating with AuNPs (Figure 3c). Nevertheless, Figure 3d exhibits a difference in the current response of the sensor with and without metals NPs against different concentration of DA, proving that AuNPs not only increase the (bio)sensor sensitivity, but, also, the selectivity toward the neurotransmitter in presence of common interferents agents, i.e., ascorbic acid (AA) and uric acid (UA).

**Figure 4:**
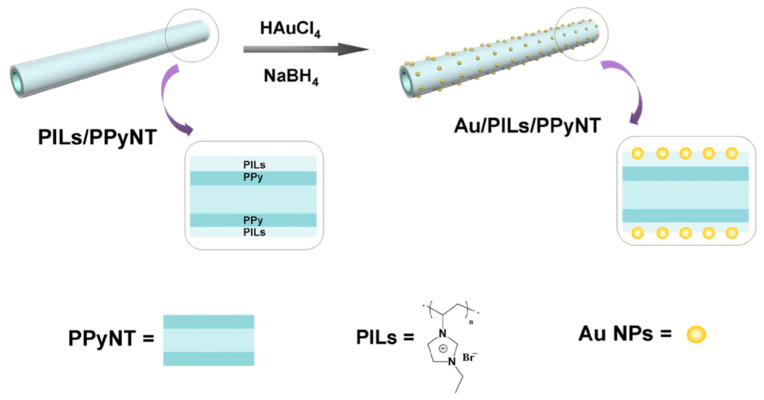
Reaction procedure for the preparation of polypyrrole nanotubes (PPyNTs)/poly(ionic liquids) (PILs)/gold nanoparticles (AuNPs). High-density and well-dispersed AuNPs could be deposited on the surface of PPyNTs/PILs by anion-exchange of PILs with Au precursor and the in situ reduction of the metal ions, due to the presence of PILs. Reprinted with permission from reference [70]. Copyright © 2017 Elsevier B.V.

A good interaction between the ICP matrix and metal NPs is essential for an optimum modification. Within this context, the study of Mao et al. evaluated poly(ionic liquids) (PILs) as linkers between AuNPs and polypyrrole nanotubes (PPyNTs), the main steps of the procedure are presented in Figure 4 [70]. The PPyNTs modified surface, displayed excellent electrocatalytic activity towards the human hormone epinephrine (EP). Hormone is not only involved in cardiac activity, also is used as a medication for the treatment of hypersensitivity reactions including anaphylaxis and hypotension derived from septic shock [71]. The EP electrochemical (bio)sensor reported a linear response in a range from 35 to 960 μM and LOD of 298.9 nM, this behavior was attributed to the PILs which promoted a high-density and uniform distribution of AuNPs on the polymer surface.

Other metals employed to increase PPy sensitive properties as electrochemical (bio)sensors are nickel and cobalt. Yang and coworkers modified the surface of over-oxidized polypyrrole nanowires (oPPyNW) with nickel hydroxide nanoflakes Ni(OH)_2_NF [72]. The electrodeposited oPPyNW on graphite electrode, were used as platform for the growth of Ni(OH)_2_NF by chemical bath method. As consequence, the hybrid oPPyNW/Ni(OH)_2_NF demonstrated an excellent performance for non-enzymatic glucose detection (LOD = 0.3 µM), associated with the efficient electrocatalytic activity and stability of both materials. Özcan et al. also fashioned a non-enzymatic glucose (bio)sensor based on an overoxidized PPy nanofibers (oPPyNFs). The ICP surface was modified with cobalt(II) phthalocyanine tetrasulfonate (CoPcTS) [73]. oPPyNFs/ CoPcTS limit of detection was 0.1 mM, within the likely glucose level in a diabetic person.

### 2.2. ICPs Modified with Carbon

Carbonaceous materials like graphene (Gr), reduced graphene oxide (rGO), graphene oxide (GO) and carbon nanotubes (CNTs) have been widely studied in the past, as result, several review papers have been published reporting different methodologies for the synthesis, processing and applications [74,75,76,77,78]. The combination of their unique characteristics with other materials, has been also explored due their promising role to enhance structural and functional properties with low manufacturing cost.

Awarded with the Nobel Prize in Physics 2010, Andre Geim and Konstantin Novoselov were the pioneers to isolate graphene [79,80]. Since then, the two-dimensional carbon allotrope, has attracted tremendous attention due to its extraordinary electrical, chemical, optical and mechanical properties, which make it a perfect candidate for the reinforcement of high-performance hybrids.

In the field of electrochemical (bio)sensors, graphene has been employed to increase the detection signal. More specifically, in 2014, Li et al. modified the structure of PPy by the incorporation of Gr, expecting a higher sensitivity in the detection of the neurotransmitters dopamine [81]. The hybrid PPy/graphene (PPy-Gr) was obtained via electrodeposition onto platinum (Pt) micro-electrodes, components of a planar microelectrode array (pMEA) fabricated by a standard micromachining process. The current response of the modified hybrid PPy-Gr and bare Pt in DA solution is displayed different behaviors. If compared with PPy-Gr, the bare Pt did not present obvious changes during the addition of DA, while the ICP hybrid recorded a well-defined stepwise curve, permitting the detection of ten times lower concentrations of DA. The LOD collected for PPy-Gr was 0.3 µM against 3 µM of DA for Pt.

Similar results were reported by Sha and coworkers who, through the surface modification of PAni with Gr, designed a non-enzymatic electrochemical (bio)sensor of urea [34]. This bioproduct is excreted by the kidneys in urine, so, kidney diseases are associated with a reduction or increase of urea concentrations in urine and in blood, respectively. Within this context, the urea sensor PAni/Gr was synthesized by electrodeposition of PAni on the surface of a glassy carbon electrode (GCE), which was previously coated with Gr via drop casting. The optimized sensor reported a LOD of 5.88 μM of urea, confirming that the modification with a carbonaceous material improved the current response of the electrochemical (bio)sensor ~4.74-fold over the unmodified PAni sensor.

A promising method for the graphene production is represented by the chemical oxidation of graphite, followed by an exfoliation and reduction processes. Taking into account that graphite is a layered material, as result of the exfoliation are obtained graphene oxide (GO) sheets with oxygen functional groups on their basal planes and edges [75]. Then, as final step, GO product is de-doped and, therefore, reconverted to its reduced state as graphene, also known as reduced graphene oxide (rGO). A work published by Wang et al. [82] proposed to convert GO to rGO by an electrochemical reduction process. For that, PEDOT was doped with graphene oxide during the ICP electrochemical polymerization, then the new hybrid was exposed to an electrochemical reduction applying a negative potential (−0.9 V for 600 s). The obtained PEDOT-rGO was used as dopamine electrochemical (bio)sensor. As was expected, the modification of PEDOT with rGO improved the electron transfer kinetics in the hybrid, leading to a highly sensitive detection of DA, with a LOD of 39 nM, even in the presence of common interferences such as uric acid and ascorbic acid. Although rGO shows better conductivity than its unreduced state, GO, some authors have taken advantages from the benefits associated to the oxidative form of graphene; for example, it can be easily dispersed in aqueous solution and the negatively charged carboxyl group in its structure acts as an excellent dopant for polymerization of conducting polymers [82,83]. In 2014, Weaver and coworkers altered a PEDOT matrix with unreduced GO, employing a electrodeposition on a GCE [35]. Results demonstrated that the electrostatic interactions between DA molecules and PEDOT-GO surface, selectively amplified the DA oxidation signal, however the LOD achieved was higher than the reported before, 83 nM.

**Figure 5:**
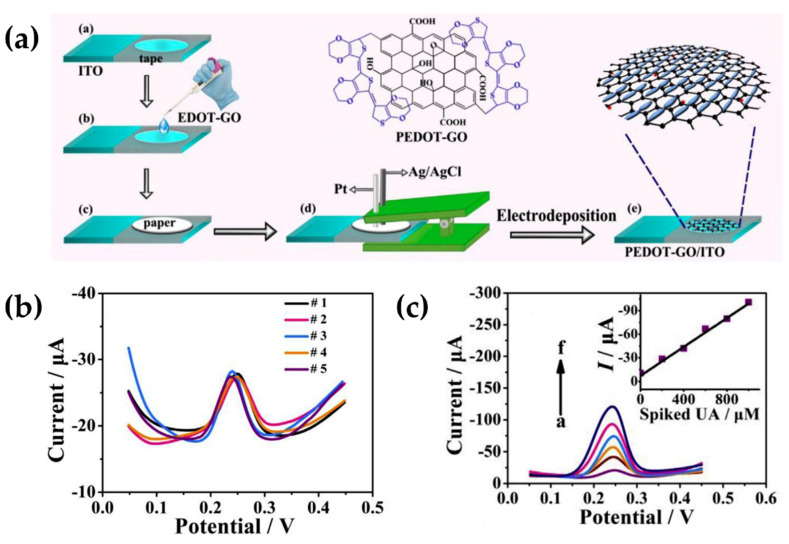
(**a**) Schematic route for the preparation of the integrated paper-based analytical device; (**b**) differential pulse voltammetry (DPV) response of the saliva sample using 5 disposable electrodes fabricated independently and (**c**) electrochemical response of saliva samples spiked with increasing concentrations of uric acid in 200 mM increments (inset calibration plot). Adapted with permission from reference [83]. Copyright © 2019 Elsevier B.V.

The structural modification of PEDOT with GO was also employed by Huang et al. to develop a paper-based analytical device, capable of detect uric acid (UA) in real human saliva, without any dilution or adjustment of the samples [83]. The modification of PEDOT was carried out during an electrochemical deposition of a mixture containing the ICP monomer and GO dispersion. A piece of paper was used as a solid electrolyte to connect the three electrodes (i.e., working, counter and reference electrode), either during the PEDOT-GO synthesis or in the UA measurements. Figure 5a reports the mentioned procedure. The benefits of the PEDOT tailored with GO and the clever idea to combine it with a paper-based analytical device, were applied for the salivary UA analysis. Figure 5b,c shows the response signal of UA oxidation in human saliva, while, the additions of UA standard solution to undiluted saliva samples in Figure 5c, confirm a linear response of the peak currents as a function of concentrations.

In order to design a material that gathers all characteristics required for an electrochemical (bio)sensor, several authors explored more than one material or modification technique at the same time. Kalloor et al. combined reduced graphene oxide and silver to modify poly (3,4-ethylenedioxythiophene) nanotubes (PEDOTNTs) [84]. The improved ICP showed better charge transfer properties, sensitivity with a LOD of 0.1 nM and selectivity to serotonin oxidation, even in the presence of ascorbic acid, uric acid and tyrosine.

**Figure 6:**
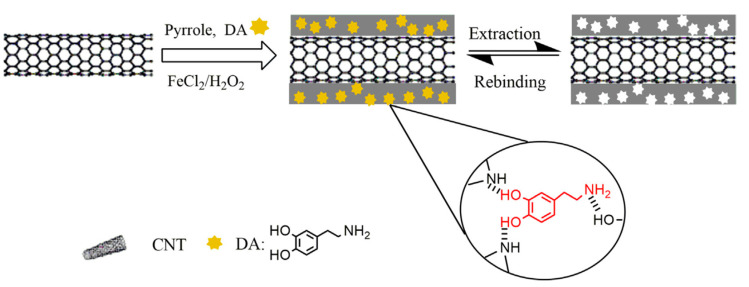
Chemical route for the preparation of molecularly imprinted polymer (MIP) polypyrrole (PPy)/ carbon nanotubes (CNTs). Reprinted with permission from reference [87]. Copyright © 2014 Elsevier B.V.

Another method employed to enhance an electrochemical (bio)sensor is the molecular imprinting technique (MIT), a system for the preparation of polymers with selective receptor sites, resulting in platforms with predetermined attraction to a specific target. For this technology, the selected monomers are polymerized around a template molecule, then, the molecule is removed from the polymer matrix leaving a stereo configuration which is used by the resulting polymer for the molecule selective recognition [36,85,86,87]. Sun and coworkers utilized MIT combined with the structural modification of PPy by graphene oxide and molecularly printed quercetin [36]. The double modification in the molecularly imprinted polymer (MIP) Ppy-Gr was designed to improve its behavior as electrochemical (bio)sensor of quercetin, a flavonoid capable of modulate enzymes activity. On one side, the GO enhanced its electrochemical sensitivity until a LOD of 4.8 × 10^−8^ M of quercetin and, on the other side, the imprinted template increased the (bio)sensor selectivity to quercetin, even in the presence of other similar flavonoids (rutin and morin). MIT technology was also used by Qian et al., but, in this case, carbon nanotubes (CNTs) were selected to increase Ppy sensitivity to DA, which was imprinted in the ICP matrix for a better selectivity (Figure 6) [87]. The modified polymer was proposed for in vivo detection of DA due its remarkable selectivity and sensitivity, achieving a LOD of 1 × 10^−11^ M.

### 2.3. ICPs Biomodified

The modification of a material by bioactive agents, such as DNA, antibodies, enzymes or microorganism, is a biomimetic approach that was studied to enhance the biochemistry of an electrochemical sensor and, consequently, improve its detection selectivity. This kind of modification is produced by common methods like physical or chemical adsorption, covalent bonding, cross-linking or entrapment of bioagents on a transducer [39,88]. Several authors have reported biomodifications in conducting polymers, which act as abiotic electroactive materials, to increase their selectivity toward bio-species.

Wei and coworkers reported a Ppy surface biomodified with the attachment of DNA-dendrimer (DDPPy) [89]. The bio-abiotic interface prevented the protein conformation change, reducing the denaturation of three salivary bio-markers for oral cancer, IL-8 protein, IL-1β protein and IL-8 mRNA. In addition, the biomodification also improved the biosensor amperometric signal, achieving a salivary bio-markers detection three orders of magnitude better than PPy without the DNA-dendrimer (LOD_DDPPy_ = 100–200 fg/mL).

**Figure 7:**
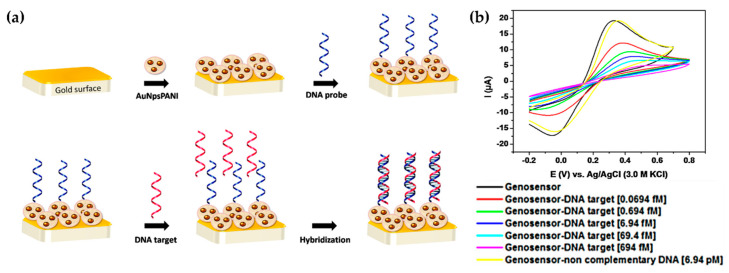
(**a**) Schematic representation of the PAni-AuNPs/DNA construction and (**b**) cyclic voltammograms or the biosensor exposed to different concentrations of recombinant plasmid containing the BCR/ABL fusion gene breakpoint cluster region- Abelson tyrosine kinase gene (DNA target: 0.0694, 0.694, 6.94, 69.4, 694 fM) and nonspecific plasmid (negative control). Adapted with permission from reference [38]. Copyright © 2016 Elsevier B.V.

In the same way, Avelino et al. utilized DNA to develop a selective biosensor of the Philadelphia chromosome associated to leukemia patients [38]. PAni was structurally modified with gold nanoparticles entrapped in its matrix for a better sensitivity. A single strand DNA (ssDNA) was captured on the PAni-AuNPs surface through electrostatic interactions. Figure 7a illustrates the biosensor synthesis route and the recognition sites by BRC/ABL fusion gene (breakpoint cluster region- Abelson tyrosine kinase gene) in leukemia (Philadelphia chromosome) as a result of their hybridization with DNA. The hybridization process leads to a gradual reduction of the amperometric response when the sensor was exposed to different concentrations of plasmodial DNA containing the BCR/ABL fusion gene, while opposite effect was collected for non-complementary plasmodial DNA. The LOD achieved was 69.4 fM of BCR/ABL fusion gene in leukemia patient samples (Figure 7b). Radhakrishnan and coworkers took advantage of the hybridization process as well, but this time, for the electrochemical recognition of DNA [37]. A PPy nanostructure was coated with PAni through an oxidative polymerization, followed by a second coating of glutaraldehyde (GA) for the immobilization of 5’-amine modified ssDNA. Detection performance was evaluated after a hybridization reaction of PPy/PANi/GA/ssDNA in the presence of methylene blue (MB) which responded to hybridized and unhybridized surfaces. The biomodified composite exhibited sensitivity and selectivity attributed to the nanostructure of PPy/PAni, a conductivity 472 times greater than conventional PPy/PAni composite and a LOD of 50 fM.

A biosensor for the electrochemical detection of acetylcholine (Ach) neuromodulator was developed by Chauhan et al. [90]. The design included a dual modification of PEDOT by a structural reinforcement with electrochemical reduced graphene oxide (rGO) and the adhesion of immobilized enzymes, acetylcholinesterase (ACh) and choline oxidase (ChO), on the surface of iron oxide nanoparticles (Fe_2_O_3_NPs). The hybrid sensor PEDOT-rGO/ACh- Fe_2_O_3_NPs exhibited a LOD and sensitivity of 4.0 nM and 0.39 μA/μM, respectively, while the average detection of ACh concentrations in the serum of healthy volunteers (*n* = 10) was 9.26 ± 2.19 nM (within the normal levels of a healthy person (i.e., 8.66 ± 1.02 nM). A similar approach was used by Bayram and Akyilmaz [39] who modified an ICP with a carbonaceous material and a bioagent. The aim of this work was the development of a microbial biosensor for the sensitive determination of paracetamol. The PAni structure was first modified by carboxylated multiwalled carbon nanotubes (cMWCNTs) during their electrodeposition on a gold working electrode, and, subsequently, the microorganism *Bacillus subtilis* was adhered on the PAni-cMWCNTs surface. In order to efficiently transform the biochemical response into a physical signal, GA was used as cross-linking agent between the recognition element (*Bacillus sp*.) and the abiotic electroactive surface, PAni-cMWCNTs. Paracetamol detection assay by PAni-cMWCNTs/ *Bacillus sp./*GA was carried out with amperometric experiments that displayed a LOD of 2.9 µM and an efficient selectivity toward paracetamol in a medium containing epinephrine, L-dopa, L-ascorbic acid, uric acid and D-glucose.

### 2.4. ICPs Modified with Other Polymers

In previous sub-sections, different modification strategies to maximize the sensitivity and selectivity of conducting polymers as Electro-BIOsensors were described. However, other properties of interest can be enhanced through the combination of ICPs with other polymers or biopolymers. An example is the work reported by Adeosun and coworkers, where the biocompatibility and conductivity of polydopamine (PDA) where complemented with PPy [91]. The outstanding biocompatibility of PDA homopolymer [92,93] and PPy-PDA copolymer [94] was previously proved, while, for the first time, in this work was studied its electrochemical response towards uric acid (UA). The biocompatibility, high conductivity and electrochemical capacitive behavior of the copolymer PPy-PDA allowed to the recognition of low concentrations of UA, with 0.1 μM as limit of detection. In addition, the biocompatible composite was used to detect UA in human serum and urine, demonstrating its potentiality in the real sample analysis.

**Figure 8:**
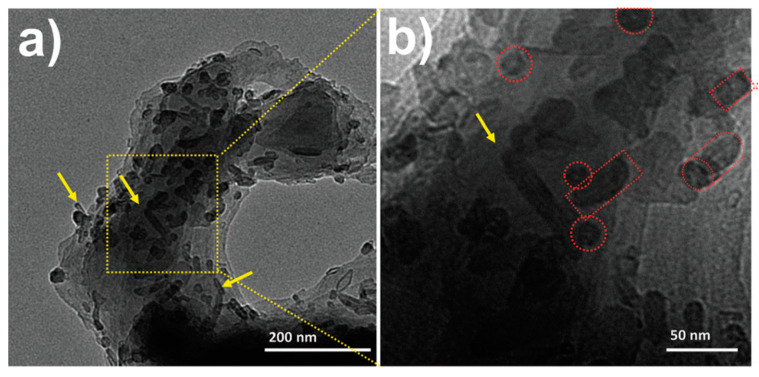
TEM micrograph of polythiophene (PTh)-*g*-(poly(ethylene glycol) (PEG)-*r*-biopolymer polycaprolactone (PCL) at (**a**) low magnification and (**b**) high magnifications. Porous spherical particles were highlighted with red circles while, rod-like structures are marked with rectangular forms and yellow arrows. Reprinted with permission from reference [96]. Copyright © The Royal Society of Chemistry 2019.

Aleman’s group [40,95] took advantage of the “grafting through” technique to structurally modify and, consequently, enhance ICPs biocompatibility through electropolymerization of polypyrrole (PPy) and a polythiophene derivative, poly(hydroxymethyl 3,4-ethylenedioxythiophene) (PHMeEDOT), with the biocompatible poly(ethylene glycol) (PEG) or the biopolymer polycaprolactone (PCL), respectively. In both cases, PPy-*g*-PEG and PHMeEDOT-*g*-PCL, cell viability assays demonstrated that the cytotoxicity of the graft copolymers was considerably reduced, if compared with the unmodified polymer. In addition, with the incorporation of non-conducting polymers that may affect negatively the electroactivity of the copolymers, the electrochemical recognition of neurotransmitters (i.e., serotonin or dopamine) was efficient, the LOD reported for PPy-*g*-PEG was 0.5 µM for serotonin and the same LOD was achieved for the detection of DA by PHMeEDOT-*g*-PCL. Employing the same technique (“grafting through”), the effects of randomly grafted PCL and PEG chains with polythiophene (PTh) were evaluated [96]. The amphiphilic character of the side chains produced a microphase separation in solution, which resulted in a PTh-*g*-(PEG-*r*-PCL) composite with two types of supramolecular structures, Figure 8 displays a TEM micrograph of the composite, where a porous spherical particles and rod-like structures were identified. Although porous PEG and/or PCL side chains growth around PTh, the biocompatible composite showed good abilities for the electrochemical recognition of NADH, with a LOD of 0.2 mM and selectivity in the presence of ascorbic acid. The graft copolymer exhibited two well defined oxidation peaks at 300 and 580 mV, corresponding to the oxidation of AA and NADH, respectively, the peak separation of 280 mV indicates that PTh grafted copolymer can be successfully used for selective detection of NADH even in the presence of AA.

Piro et al. modified the surface of the electropolymerized PEDOT with carboxylic acid PEG, employing the non-conducting polymer as a cross-linker between the PEDOT and the enzyme glucose oxidase (GOD) [97]. The enzyme was attached to carboxylic acid PEG forming peptide bonds between the amine groups of GOD and the carboxylic acid groups of PEG. Afterwards, PEG-GOD was entrapped within PEDOT films electrogenerated on glassy carbon electrodes (GCE). Amperometric assay in the presence of glucose and ferrocene as mediator, indicated that the biosensor PEDOT/PEG-GOD possessed good sensitivity up to 22 mM, quite similar to the unmodified GOD electrode (i.e., PEDOT/GOD). However, opposites results were obtained from stability assays indicating that the PEG incorporation in PEDOT surface increased the biosensor stability against time.

**Figure 9:**
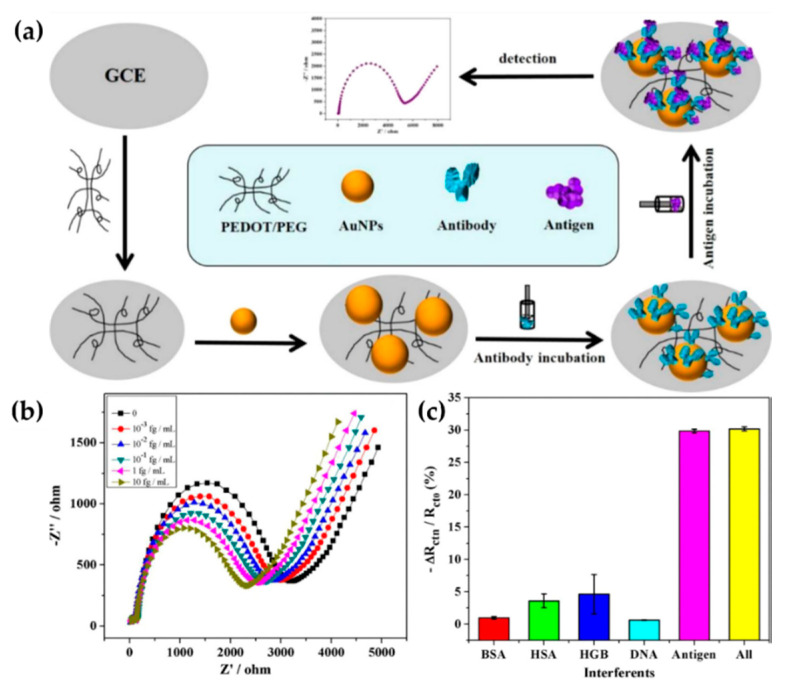
(**a**) Schematic illustration of of α-fetoprotein (AFP) biosensor synthesis; (**b**) impedance spectra corresponding to the biosensor with different antigen concentrations (0.01-M PBS, pH 7.4), curves from inner to outer represent 10 fg/mL, 1 fg/mL, 10^−1^ fg/mL, 10^−2^ fg/mL, 10^−3^ fg/mL AFP antigen, respectively; (**c**) responses of the AFP biosensor to bovine serum albumin (BSA) (1.0 nM), human serum albumin (HSA) (1.0 nM), hemoglobin (HGB) (1.0 nM), DNA sequence (1.0 nM), AFP antigen (1.0 fg/mL) and a mixture of all the above substances, respectively. Adapted with permission from reference [41]. Copyright © 2016 Elsevier B.V.

Another example of the benefits associated to the combination of PEDOT and PEG is represented by the work published by Cui and coworkers in 2016 [41]. In this case, PEDOT matrix was structurally modified with a PEG derivative, 4-arm PEG terminated with thiol groups. A second modification was carried out with AuNPs introduced to the copolymer surface through their interaction with the thiol groups. AuNPs provided support for the immobilization of α-fetoprotein (AFP) antibody, a vital tumor biomarker for liver cancer. The synthesis route of PEDOT-PEG/AuNPs-AFP is displayed in Figure 9a. EIS was employed to examine hybrid’s biosensing performance after its incubation in target AFP antigen solution at different times, as can be observed in Figure 9b. EIS experiments proved that PEDOT electroactivity in the hybrid, provided high sensitivity to AFP antigen. Higher the antigen concentration, lower the charger transfer resistance, with a LOD of 0.0003 fg/mL (S/N = 3). On the other hand, antibody immobilization on AuNPs permitted an excellent selectivity toward AFP antigen, results displayed in Figure 9c compare the signal response of the hybrid in solutions containing bovine serum albumin (BSA), human serum albumin (HSA), hemoglobin (HGB) or single strand DNA sequence. As can be observed, the hybrid showed a negligible signal response in all cases with the exception of solution containing the target AFP antigen, either separated or combined with the other substances. Besides cross-linked PEDOT and AnNPs-AFP, 4-arm PEG apported hydrophilicity to the hybrid and, therefore, it exhibited good anti-fouling ability which allowed the detection of target AFP in 10% (*v/v*) human serum samples.

Although ICPs have a remarkable sensitivity towards several biomolecules due their structure, their rigid backbone also causes their man handicap, a limited processability as result from the lack of mechanical properties, an essential quality for polymers been employed in biomedical implants or devices [98,99]. Hence, fashioning a conducting biomaterial with acceptable processability represent one of the major challenges in the biosensing field. Aleman’s group fashioned a strategy to overcome this limitation [100]. PEDOT NPs where reinforced by isotactic polypropylene (i-PP), which added mechanical integrity to the composite, with a copolymer ratio of 40:60 i-PP:PEDOT. However, in the same work the null electroactivity of i-PP was introduced by its structural modification trough PEDOT NPs with a copolymer ratio of 60:40 i-PP:PEDOT. The composites iPP-PEDOT 60% and i-PP-PEDOT 40% reveled new and better mechanical or electrochemical properties, respectively, in both cases affected by the interfacial adhesion between their components. As electrochemical (bio)sensor, iPP-PEDOT sensed the growth of Gram-negative and Gram-positive bacteria through the extracellular oxidation of nicotinamide adenine dinucleotide (NADH) produce by prokaryotic cells.

A detailed description of non-conducting polymer modified for its used as Electro-BIOsensors will be explain in the following section.

## 3. Electro-BIOsensors Including Non-Conducting Polymers

*Plastics are engineered to last!* Plastics manufacturers generally introduce extra stabilizing chemicals to give their products longer life, which unfortunately decrease their compatibility with the environment and the human health. With society’s ever-increasing focus on protecting the environment, there is a new emphasis on designing plastics that will disappear much more quickly or that are directly coming from nature. The so-called "environmentally friendly" plastics fall into three types: bioplastics made from natural materials such as corn starch, biodegradable plastics made from traditional petrochemicals, which are engineered to break down more quickly and eco/recycled plastics, which are simply plastics made from recycled plastic materials rather than raw petrochemicals. We examine each of these in their newest utilization in biosensors based on electrochemical detection, mainly as matrix modified to improve conductive properties.

### 3.1. Bioplastics and Biodegradable Plastics

**Figure 10:**
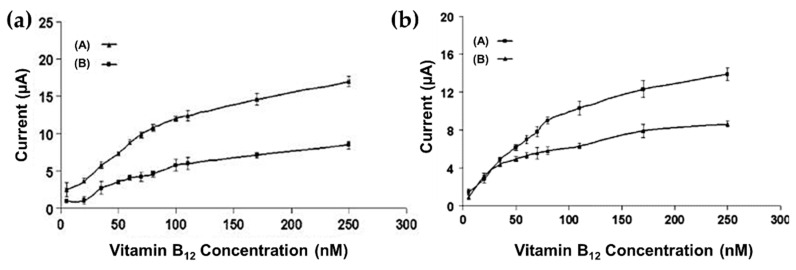
(**a**) Effect of vitamin B12 concentration on reduction peak currents of Co(II) to Co(I) using (A) single walled carbon nanotube (SWCNT)–chitosan modified PGE, (B) chitosan modified PGE at pH 2.0; (**b**) The effect of vitamin B12 concentration on reduction peak currents of Co(II) to Co(I) using (A) SWCNT–chitosan modified PGE; (B) chitosan modified PGE at pH 5.0. Adapted with permission from reference [108]. Copyright © 2011 Elsevier B.V.

Chitosan (CS) is a biopolymer (a polysaccharide) obtained by the partial deacetylation of chitin [101,102], with excellent nontoxicity, biocompatibility, biodegradability, multiple functional groups, pH-dependent solubility in aqueous media, cheapness and a susceptibility to chemical modification [103,104,105,106]. One of the most innovative application of chitosan and its derivatives is the development of specific sensors and electrochemical devices due to the chemical and electrical features, the interesting mechanical and biologic properties of the chitosan-based materials [107]. Although chitosan may present useful characteristics alone, many applications explore its use through chemical modifications or in composites, leading to materials that may present mixed characteristics or, in some cases, better performance due to synergic effects. Kuralay et al. [108] reported an interesting work on the development of single walled carbon nanotube (SWCNT)–chitosan modified disposable pencil graphite electrode (PGE) for the electrochemical monitoring of vitamin B12. The device aimed to achieve a signal enhancement of the analyte in comparison to chitosan modified disposable pencil graphite electrode. The selected molecule (vitamin B12) is a corrin based cobalt complex which is important in human physiology because its deficiency causes pernicious anemia and neuropathy [109,110]. It can be detected by electrochemical techniques [111] due to the redox chemistry centered on the cobalt atom: vitamin B12a (with Co(III)) can be reduced reversibly to vitamin B12r (with Co(II)), and be further reduced to vitamin B12 s (with Co(I)), all in aqueous media [111]. The SWCNT–chitosan modified PGE was prepared in a one-step procedure: the incorporation of SWCNTs into the positively charged polymer matrix was carried out by immersion of PGE in SWCNT–chitosan mixture and chitosan solution. The electrochemical response of SWCNT–chitosan modified PGE was compared with the references response of chitosan modified PGE for vitamin B12 analysis. A signal enhancement was obtained for the reduction of cobalt redox couples in the structure of vitamin B12, using SWCNT–chitosan modified PGE at low potentials due to the catalytic activity of SWCNT [108]. Different values of LOD were found by changing the pH of the solution. In particular the LOD increased from 0.89 nM at pH 2.0 (concentration range interval of 5nM and 100 nM, Figure 10a) to 2.1 nM at pH 5.0 (concentration range interval of 5nM and 80 nM, Figure 10b) [108].

Devnani et al. synthesized graphene-chitosan (GRP-CHIT) glassy carbon electrode (GCE) for the sensitive and selective electrochemical determination of Noradrenaline (NA), using cyclic voltammetry (CV) and square wave voltammetry (SWV) as electrochemical techniques [42]. NA is a catecholamine derivative, secreted and released by adrenal glands, critically linked to mental diseases, heart failure, diabetes [112,113]. NA is an electroactive compound, but, unfortunately, ascorbic acid and uric acid share similar redox potential and thus interfere with its analysis [114]. In order to improve sensitivity and achieve selectivity, a chemically modified electrodes using nanomaterials as electron transfer mediators were fabricated in this work. Various nanocomposites consisting of either metal-metal oxide, mixed metal oxides, polymers mixed with metal or metal oxides or carbon nanotubes mixed with polymers, metals or metal oxides have attracted attention as active materials for electrochemical sensing [115]. In this study, the easily fabricated GRP- CHIT/GCE (drop-casting of GRP-CHIT solution over the surface of GC modified), showed enhancement in current response, while electrochemical impedance spectroscopy (EIS) showed reduction in charge transfer resistance at the modified sensor, whose applicability was tested in human blood serum. Limit of detection (LOD) and limit of quantification (LOQ) were obtained as 19.7 nM and 65.8 nM. Ascorbic acid and uric acid were found not to pose interference to the NA detection.

**Figure 11:**
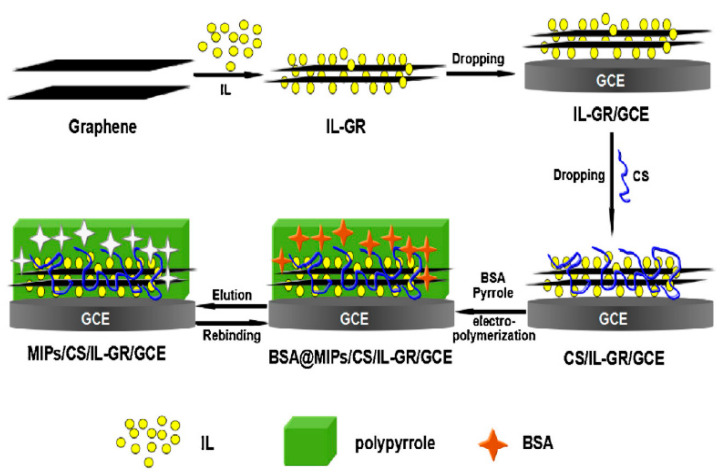
Schematic diagram of the preparation procedure of the molecular imprinted electrochemical sensor. Adapted with permission from reference [43]. Copyright © 2015 Elsevier.

In a novel study, Xia et al. [43] have prepared an efficient molecularly imprinted chitosan/ionic liquid–graphene modified glassy carbon electrode (MIPs/CS/IL–GR/GCE) using bovine serum albumin as the template molecule and PPy electropolymerized onto the surface of CS/IL–GR/GCE as substrate material. The idea of the authors is very interesting because combines molecular imprinting and electrochemical sensor into molecularly imprinted electrochemical sensor (MIECS), characterized by high selectivity and sensitivity. The authors, for the first time, used MIECS for the imprinting of protein. CS has been used as an immobilization matrix in order to improve the biocompatibility of the interface [116], while graphene (GR) and ionic liquid (IL) have been selected to construct the sensing interface. The unique physicochemical properties of GR [117,118] and the high ionic conductivity, wide electrochemical windows and good chemical stability [119,120] of IL allows to achieve excellent electrochemical catalytic ability for electrochemical detection [121,122]. Moreover, ILs can interact with protein, in order to facilitate the transfer of protein into the liquid phase and optimize the imprinted amount, and also can improve the dispersibility of graphene, which is favorable for the improvement of the electrochemical performance [123]. The detailed preparation procedure of the MIPs/CS/IL–GR/GCE was illustrated in Figure 11.

**Figure 12:**
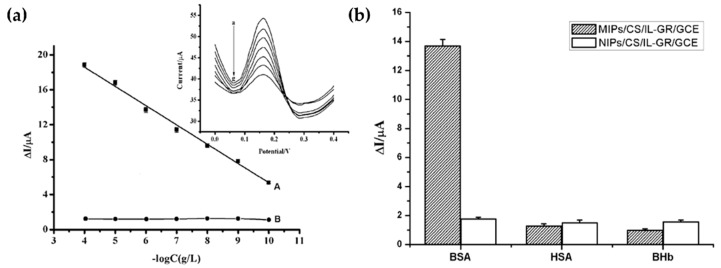
(**a**) Calibration curve of the prepared (A) molecularly imprinted (MIPs)/chitosan (CS)/ionic liquid–graphene (IL-GR) modified glassy carbon electrode (GCE) (MIPs/CS/IL–GR/GCE) and (B) MIPs/CS/IL–GR/GCE for different concentrations of BSA in PBS containing 0.10-mM [Fe(CN)_6_] ^3−/4−^ (pH 7.0) (insert: the DPVs with different concentrations of bovine serum albumin (BSA) on MIPs/CS/IL–GR/GCE); (**b**) selectivity of MIPs/CS/IL–GR/GCE and MIPs/CS/IL–GR/GCE for BSA, human serum albumin (HSA) and bovine hemoglobin (BHb). The concentration of each protein is 1.0 × 10^−6^ g/L. Reprinted with permission from reference [43]. Copyright ^©^ 2015 Elsevier B.V.

As shown in Figure 12a (insert), the DPV peak currents of the MIPs/CS/IL–GR/GCE decreased with the increment of BSA concentrations and a linear relationship between the changes of current response (ΔI) and the logarithms of BSA concentrations has been observed. The detection limit (2 × 10^−11^ g/L) was smaller than those obtained from other molecularly imprinted sensors [124,125,126]. Human serum albumin and Bovine hemoglobin (BHb) were used as control proteins as shown in Figure 12b where it can be observed that the ΔI on MIPs/CS/IL–GR/GCE toward BSA was the highest, which was 10.6 and 14.2 times of that toward HSA and BHb, respectively.

**Figure 13:**
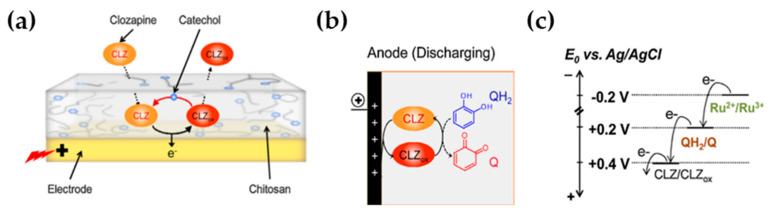
Clozapine (CLZ) as an oxidizing mediator in the catechol-modified chitosan system. (**a**) Schematic of the system with the diffusing CLZ; (**b**) continuous oxidation of CLZ in the presence of catechol (Q) reduction; (**c**) CLZ acts as an oxidizing mediator of QH_2_ and Ru^2+^ as a reducing mediator regenerating the Q. Electrochemical potential bar represents standard reduction potential of Ru^2+^, Q and CLZ. Reprinted with permission from reference [44]. Copyright ^©^ 2015 © 2014 Elsevier, Ltd.

Another interesting work based on chitosan polymer utilization and related to the employment of electrochemical detection in the neuroscience field was published from Ben-Yoav et al. [44]. They studied the functionality and detection limit of clozapine (CLZ) (a second-line antipsychotic for schizophrenia treatment, for patients who are unresponsive to other antipsychotics) and characterized the selectivity with respect to the CLZ principal metabolite norclozapine (NorCLZ). The goal of this study is the utilization of microfabrication technology for the development of on-chip electrochemical microsystems, where the sensing electrodes are integrated directly onto the microchip. Figure 13 shows the biofabricated catechol-modified chitosan redox cycling system. CLZ (E_0_ = +0.4 V) can diffuse within the chitosan film and the grafted catechol moieties can be interconverted between oxidized (Q) and reduced (QH_2_) forms (E_0_ = +0.2 V). CLZ is reduced by the grafted QH_2_ moieties and it is electrochemically re-oxidized at the electrode (Figure 13a–c). A continuous cycle of CLZ reduction in the presence of catechol followed by CLZ re-oxidation results from this use of CLZ as an oxidizing mediator (Figure 13b) and it is responsible of the increase of the total charge transferred by CLZ oxidation, amplifying the generated electrochemical current and improving the signal-to-noise ratio. To recover the redox cycling system to the reduced state, negative potential is applied in the of the presence of a reducing mediator, HARu (Ru^2+/3+^, E_0_ = −0.2 V).

Electrochemical investigations, carried out with CLZ in the presence and in the absence of the catechol-modified chitosan amplification system, showed that CLZ oxidation peak recorded with the modified electrode was 3-fold higher than the peak current density with the unmodified bare electrode. The modified electrode yielded a measured detection of 0.1 μg/mL CLZ, compared to 3.26 μg/mL for the unmodified electrode.

Polylactic acid (PLA) is one of the biodegradable biopolymers that have recently gained attention in bioanalysis areas due to its special characteristics, including biocompatibility and sustained release properties [127,128]. Recently, highly stable PLA-AuNPs were proven to significantly improve both surface area and particle size of modified electrodes [45]. Taking into account the possibility to obtain a stabilization of AuNPs between the carboxylic groups of the PLA polymer matrix [129], Hajian’s group [45] fabricated a PLA-stabilized AuNPs-based electrochemical sensor. In particular, a screen-printed carbon electrode (SPCE) acting as a disposable sensor for detection of DNA hybridization was modified by drop cast with homogenous AuNPs and PLA-stabilized AuNPs solution (AuNPs/SPCE and PLA-AuNPs/SPCE, respectively. Self-assembled monolayer technique based on thiol-AuNPs bonding process was used for bonding probe single-stranded DNA (ssDNA) on the surface of AuNPs and PLA-AuNPs modified electrode by linking AuNPs to thiol groups (DNA-AuNPs/SPCE and DNA/PLA-AuNPs/SPCE, respectively). The performance of the developed biosensor was investigated by CV analysis in the presence of methylene blue (MB) redox indicator, incorporated at DNA surface.

**Figure 14:**
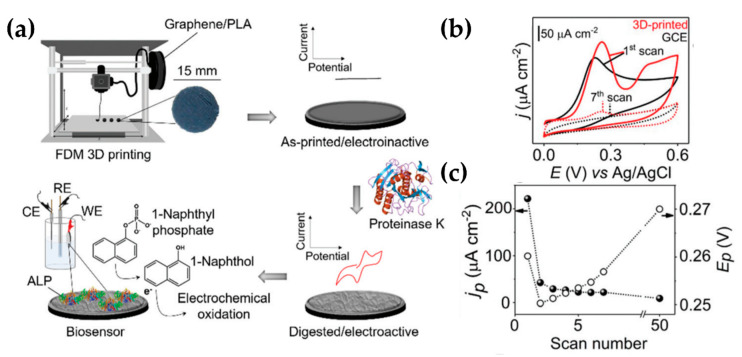
(**a**) Representation of the 3D-printed graphene/PLA electrodes’ fabrication, digestion/activation and application: first, coin-shaped electrodes from the graphene/PLA composite filament are 3D-printed with a fused deposition modeling printer. After proteinase K-mediated PLA digestion, the electrodes’ surface becomes eroded and electroactive. The resulting activated surface is used to immobilize alkaline phosphatase (ALP) enzyme via adsorption. ALP catalyzes the conversion of 1-naphthyl phosphate into 1-naphthol, which is electrochemically oxidized at the surface of 3D-printed electrodes. Electrooxidation of 1-naphthol at the digested 3D-printed sur- faces; (**b**) CVs performed on the activated 3D-printed electrodes and glassy carbon electrode (GCE) in the presence of 1-naphthol (60 μM); (**c**) progression of maximum current density ( *jp*) and peak potential (*Ep*) with the number of scans, on activated 3D-printed surfaces. Reprinted with permission from reference [46]. Copyright © The Royal Society of Chemistry 2019.

3-D printing has entered the field of electrochemistry with the advent of conductive printable materials and the fabrication of 3D-printed electrodes for sensors [46,130]. Recently, Pumera´s group [46] reported 3D-printed electrodes made from commercial graphene/polylactic acid (PLA) composite, to detect picric acid and ascorbic acid [131]. The major disadvantage of this composite is connected with the high bulk content of the thermoplastic polymer in the final electrodes after 3D printing that hinders the conducting and electroactive carbon-based part from exposition to the electrolyte. These composites need to be activated by the organic solvent method or electrochemical pretreatment without disrupting their structural and mechanical properties. In a novel work of the same group, they proposed an ecofriendly solution of the before commented drawback, by using a highly controllable biocatalytic process. The biodegradability property of the PLA was suited to conduct a partial digestion of the 3D-printed electrodes [130]. The abiotic hydrolysis of PLA is significantly accelerated by extracellular enzymes such as proteinase K produced by microorganisms naturally occurring in a soil environment. The authors achieved a controlled patterning of the 3D printed electrode and also explored the application of the digested surfaces as biosensors, showing that these enzymatically sculptured 3D-printed objects can serve as immobilization platforms for biomolecules and electro- chemical transducers (Figure 14a). More in details, they evaluated the biosensing capabilities by immobilizing an enzyme via physical adsorption and utilizing 1-naphthol as an enzymatic redox product. The enzyme, alkaline phosphatase (ALP) whose activity, in routine clinical laboratory tests, is usually monitored for the diagnosis and therapeutic observation of bone and hepatobiliary diseases [132], can convert a specific electroinactive substrate, 1-naphthyl phosphate, into the electroactive 1-naphthol. The CVs performed on the activated 3D-printed electrodes and glassy carbon electrode (GCE) in the presence of 60-μM 1-naphthol and the progression of maximum current density (*j_p_*) and peak potential (*E_p_*) with the number of scans, on activated 3D-printed surfaces are reported in Figure 14b,c.

Polyvinyl alcohol (PVA) is a synthetic water-soluble biopolymer, a hydrogel frequently used in biomedical applications which possesses good mechanical and thermal properties, good transparency and resistance to oxygen permeation and it is an ideal material for enzyme immobilization because of its nontoxic nature and good bio-compatibility [133]. PVA also offers unique characteristics such as excellent gel-forming properties and good film-forming ability, which make it an excellent candidate for enzyme immobilization. The entrapment of glucose oxidase (GOD) employed for the detection of glucose, was carried out on silica sol−gel/PVA composite films [134], endowed with a great amount of hydroxyl groups and providing a bio-compatible microenvironment for the encapsulation of GOD or on a PVA-based support chemically cross-linked with glutaraldehyde, prepared with silicate sol−gel and alumina sol−gel [135]. Both types of sol−gel methods were brittle, but with the addition of PVA increased the mechanical strength. The main drawback associated is due to the compaction and low-conductivity of such polymer membranes that make difficult for the substrate to infiltrate into the enzyme membrane and for the electrons to effectively transfer between the enzyme membrane and the electrode. To improve on this, research groups have employed PVA and other polymers to form composite matrices with NPs to enhance the electron transport to the electrode surface while retaining their original beneficial physical properties.

Lad et al. [136] developed an amperometric glucose enzyme electrode by the immobilization of glucose oxidase (GOD) in a PVA composite material based on PVA and partially prehydrolyzed tetraethyl orthosilicate (pphTEOS) on the surface of “in-house” fabricated graphite electrodes. For comparison, silver and gold nanoparticles (Ag/AuNPs) embedded in the PVA-pphTEOS matrix was prepared through a novel method via sol−gel process based on the in situ chemical reduction of Ag or Au ions using PVA as a reducing agent and stabilizer. The successful incorporation of Ag and AuNPs ranging from 5 to 7.5 and 4.5−11 nm, respectively. The limit of detection for the enzyme electrode was estimated to be 0.35 mM at a signal-to-noise ratio (S/N) of 3.

**Figure 15:**
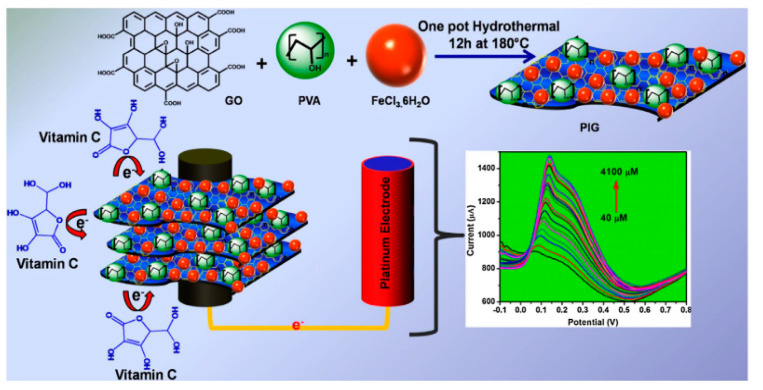
Schematic representation of synthesis and detection of vitamin C using graphene-iron oxide-polyvinyl alcohol (PIG). Reprinted with permission from reference [47]. Copyright © 2018 Elsevier B.V.

In a recent work [47], nanocomposites of graphene-iron oxide-polyvinyl alcohol (PIG) were synthesized via simple one-pot hydrothermal method and were used in the modification of pencil graphite electrode, for developing an electrochemical sensor for ascorbic acid, i.e., vitamin C detection. The presence of PVA polymer brings improvements as increased electrocatalytic activity and large surface area. The excess presence of hydroxyl group provides a large number of electrons during the process. PVA also supports the composite for uniform coating on the electrode surface and also acts as a mediator for facilitating electron transfer. Figure 15 reports the schematic representation of the detection of vitamin C shows: *i*) the oxidation of ascorbic acid by releasing electron at the PIG modified electrode, *ii*) a current flows through the external circuit which corresponds to oxidation current and, finally, *iii*) the increase in the concentration of the analyte that gradually increases the current response obtained for every spike. At optimized condition, the PIG modified electrode shows a high linear range (40 μM–4100 μM), low limit of detection (0.234 μM) and higher sensitivity (1597.03 μA cm^−2^ mM^−1^). The PIG material also provides a good stability towards detection of vitamin C.

### 3.2. Eco/Recyclable Plastics

The practice of medicine has entered an age of rapid progress, and plastics are at the core of medical innovation because they are flexible enough to fit into the smallest places, and they are durable enough to withstand decades of wear and tear. Plastics are the primary choice for prostheses, for long-term implanted medical devices and plastic packaging helps keep medicine and medical devices safe and free of contamination. The drawback connected with the utilization of plastic is the environmental contamination which they are responsible of. Many industrial and academic efforts are devoted to increase the acceptance of all plastic products at all recycling facilities and continue to innovate to make it easier.

**Figure 16:**
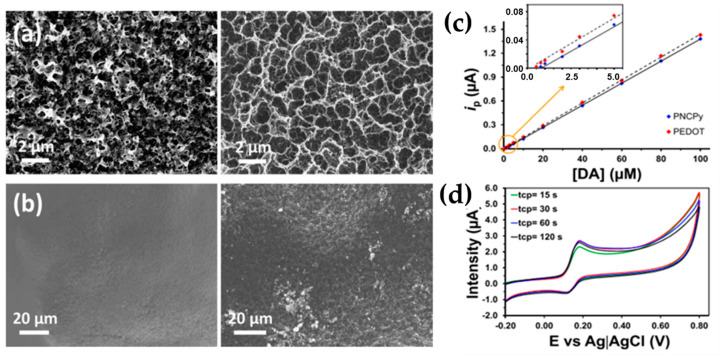
SEM micrographs of (**a**) poly(3,4-ethylenedioxythiophene) (PEDOT) and (**b**) poly(N-cyanoethylpyrrole) (PNCPy) before (left) and after (right) plasma treatment using t_cp_ = 2 min. (**c**) DA detection limit of PEDOT- and PNCPy-modified glassy carbon electrodes (GCEs) with cold- plasma treatment, as obtained from the standard addition of 10 μL of DA to 10 mL of 0.1-M PBS. Anodic peak intensity (i_p_) was determined by CV using a scan rate of 50 mV s^−1^; (**d**) control voltammograms of 100 μ-M DA in 0.1-M PBS at cold-plasma treated PEDOT-modified GCE prepared using different t_cp_ values. Scan rate: 100 mV/s. Reprinted with permission from reference [48]. Copyright © 2016 Elsevier B.V.

Very recently, some scientist are focusing their attention to the study of advanced materials based on recyclable polymers used as biosensor and employing an electrochemical detection [48,49]. Fabregat et al. [48] reported on the application of cold plasma technologies as a very simple and effective modification technique for the preparation of dopamine electrochemical sensors, fabricated using not only electrochemically active CPs, as for example poly(3,4-ethylenedioxythiophene) (PEDOT) and poly(N-cyanoethylpyrrole) (PNCPy), but also conventional insulating and electrochemical inert plastics, as polypropylene (PP) and polystyrene (PS). They modified the surface of the polymers in a room-temperature air-discharge plasma and showed that the electrochemical response of the plasma-functionalized materials was strongly enhanced. The application of air–plasma to polymeric films coating glassy carbon electrodes (GCEs) during only 1–2 min was enough to fabricate sensors with resolution and sensitivity similar to those achieved through sophisticated chemical modifications. The plasma exposure induced functionalization of the polymeric surface, due to hydrogen separation from polymeric chains, free radical creation and, finally, interaction of the radicals with oxygen and nitrogen. New functional groups were incorporated into the polymer surface and it was found that the nature of reactive species formed strongly depends on the chemical structure of the polymer. The presence of reactive species at the surface is responsible of the transformation of DA to its oxidized form DA-o-quinone (DQ) and their nature affects the capacity of the surface to exchange electrons with the surrounding environment. Figure 16c evidences a linear behavior in the whole interval of examined DA concentration (from 0.5 to 100-μM DA). The detection limit expressed as 3.3 σ/S, where σ and S is the standard deviation of the response and the slope of the calibration curve for DA concentrations ranging from 0.5 to 5 μM (inset of Figure 16c) is 140 and 750 nM for PEDOT and PNCPy, respectively. These values are significantly lower than those obtained for non-functionalized samples [68,69], evidencing an improvement not only in the resolution (especially for PNCPy), but also in the sensitivity.

As shown by SEM micrographs (Figure 16a), the compact morphology of PEDOT and PNCPy untreated by plasma, was transformed into porous networks after plasma application (Figure 16a,b, right). In the case of PEDOT (Figure 16a), abundant pores were created which facilitates the diffusion of DA molecules and, therefore, the effectivity of the electron transfer process. For PNCPy, the extremely compact surface becomes porous after plasma treatment, which is consistent with enhancement of the electrochemical response. As a probe of concept, the authors applied the same physical treatment onto the surface of an electrochemically inactive polymer, such as low density polyethylene (LDPE), transforming it into an electroactive material suitable for the fabrication of very cheap electrochemical sensors. For this purpose, LDPE sensors were manufactured by solvent casting onto GCE substrate and electrochemical assays (CV) showed that LDPE is not able to detect such neurotransmitter without cold plasma treatment, but, in contrast, the voltammogram recorded using an identically fabricated electrode, but applying a cold-plasma treatment during only 1 min, shows a sharp oxidation peak corresponding to the DA peak. By comparing the sensitivity and resolution obtained for the determination of dopamine, ascorbic acid and uric acid using plasma treated LDPE-modified electrodes with those achieved using PEDOT- and PNCPy-modified electrodes, the authors found that the electrochemical peaks of the three analytes are similarly for the three plasma treated polymer-modified electrodes. Thus, the performances of the treated LPDE is similar to that of treated conductive polymers

A step forward in the functionalization of a such LDPE-CGE electrode was presented by the same group in another work [49], where the applicability of the plasma-treated low-density polyethylene (LDPE) as an insulating and electrochemically inert polymer acting as mediator for the fabrication of enzymatic glucose sensors was explored. The LDPE based biosensor was compared with a PEDOT based, significantly more expensive and sophisticated than LDPE. The goal of this study was the investigation of the effectiveness of plasma-functionalized polymers as mediators in glucose sensors, by promoting the electrochemical communication between the Glucose oxidase (GOx) and the substrate, and also the evaluation of the oxidation of interferents like DA, UA and AA, affecting the glucose detection. Thus, such coupling has been used to propose a bifunctional biosensing platform to detect simultaneously glucose and DA.

**Figure 17:**
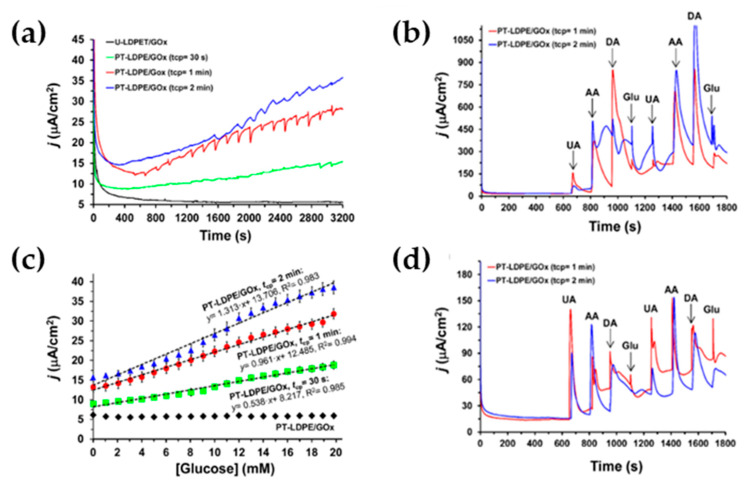
(**a**) Current–time plots for the untreated low-density polyethylene (U-LDPE)/glucose oxidase (GOx) and treated low-density polyethylene (PT- LDPE)/GOx (t_cp_ = 30 s, 1 and 2 min) upon the successive addition in 0.1-M PBS of 1 mM glucose; (**b**) current–density response versus glucose concentration for the three sensors mentioned above. Error bars indicate standard deviations for five measurements using independent electrodes. The calibration curve equation is also displayed. Current–time plots for the PT-LDPE/GOx sensors (t_cp_ = 1 and 2 min in red and blue, respectively) upon the successive addition in 0.1-M PBS of: (**c**) 1 mM glucose, 1 mM uric acid (UA), 1 mM ascorbic acid (AA) and 1 mM dopamine (DA); (**d**) 1 mM glucose, 0.1 mM UA, 0.1 mM AA and 0.1 mM DA. Polarization potential: 0.50 V versus Ag|AgCl. Reprinted with permission from reference [49]. Copyright © 2017 WILEY-VCH Verlag GmbH & Co. KGaA, Weinheim.

The authors found a correlation between the time of the plasma treatment and the electrochemical response. In details, as reported by Figure 17a,b, they compared electrodes untreated (*U*-LDPE/GOx) and treated (PT-LDPE/GOx) by plasma at different time (t_cp_ = 30 s, 1 and 2 min), confirming that LDPE electrode is not able to detect the oxidation of the glucose to gluconolactone, being an electrical insulator and hindering the transfer of electrons from GOx to the GC substrate. On the other hand, the samples treated by plasma detected the glucose with a LOD and a sensitivity increased from 0.9 to 1.7 mM and from 0.54 to 1.31 μA· cm^−2^ mM^−1^, respectively, when t_cp_ grows from 30 s to 2 min. An opposite behavior was found for PEDOT- based electrodes, in which the electron transfer was more efficient for *U*-PEDOT/GOx than for PT-PEDOT/GOx, the efficiency of the latter decreasing with increasing t_cp_. It suggests that because of the modification coming from the plasma treatment, the reactive species affect the electron delocalization decreasing the electronic conduction for PT- PEDOT (impeded π-electron delocalization) and increasing the electronic performance of PT-LDPE. The role of the polymers in enzymatic glucose sensors is crucial for an of efficient electrical communication between the GOx and the GC surface. The chronoamperometric response to the glucose and interferents injection of PT-LDPE/GOx sensors was also investigated for systems with mediators based PT-LDPE films produced using t_cp_ = 1 min (Figure 17c,d). The peaks associated to the injection of glucose, DA, UA and AA are clear resolved and exhibit very different current densities, evidencing that PT-LDPE/GOx with t_cp_ = 1 min act as efficient bifunctional sensors for the selective detection of glucose and DA.

In a fascinating investigation [50], a flexible platinum electrode was fabricated onto Bio-PET surfaces, with the aim to detect biomarkers related to Parkinson’s disease. This work is of particular interest because it tried to solve a problems connected with this critical disease affecting slightly more than 1% of the population over 65 years old and approximately 0.3% of the world population [137], whose diagnosis is quite difficult due to the fact that the exact causes of the disease are still unknown. Early identification of this disease is a challenge and strongly linked to the need to detect specific biomarkers as protein deglycase DJ-1, encoded by the gene PARK7 (PARK7/DJ-1). DJ-1 was reported as a potential biomarker playing a significant role in antioxidative defense, protecting neurons from oxidative stress [137] and preventing from Parkinson’s disease. It is also essential to notice that the decrease of DA is one of the leading causes of Parkinson’s disease symptoms [138].

**Figure 18:**
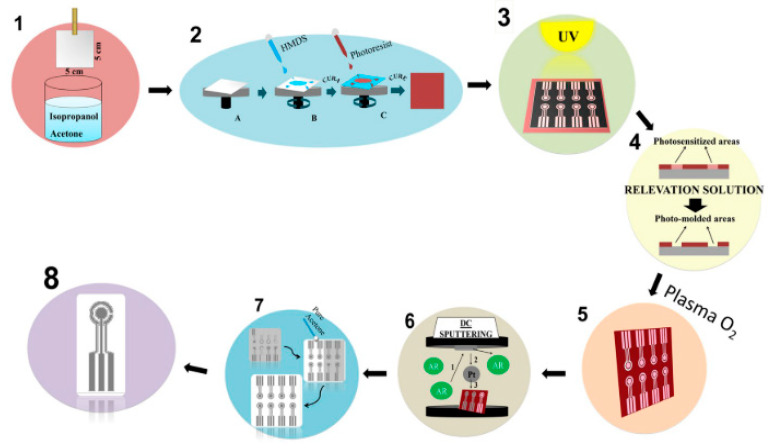
(**1**) Bio-polyethylene terephthalate (PET) pretreatment; (**2**) photoresist deposition on substrate; (**3**) exposure to ultraviolet radiation for electrode delimitation; (**4**) removal of the sensitized photoresist with the developer solution, followed by washing and drying; (**5**) patterned substrate treatment with O_2_ plasma; (**6**) substrate treatment with O_2_ plasma; (**7**) removal of excess coating and washing material; (**8**) individually cut flexible platinum electrodes. Reprinted with permission from reference [50]. Copyright © 2020 Elsevier B.V.

The microfabrication of platinum electrodes was carried out by conventional photolithography and the main steps are reported in Figure 18. Bio-PET represents an eco-friendly alternative to traditional PET because its production comes from biomass, different from petroleum derivatives of the conventional version of PET. However, this bio-based polymer presents similar characteristics of flexibility and malleability.

**Figure 19:**
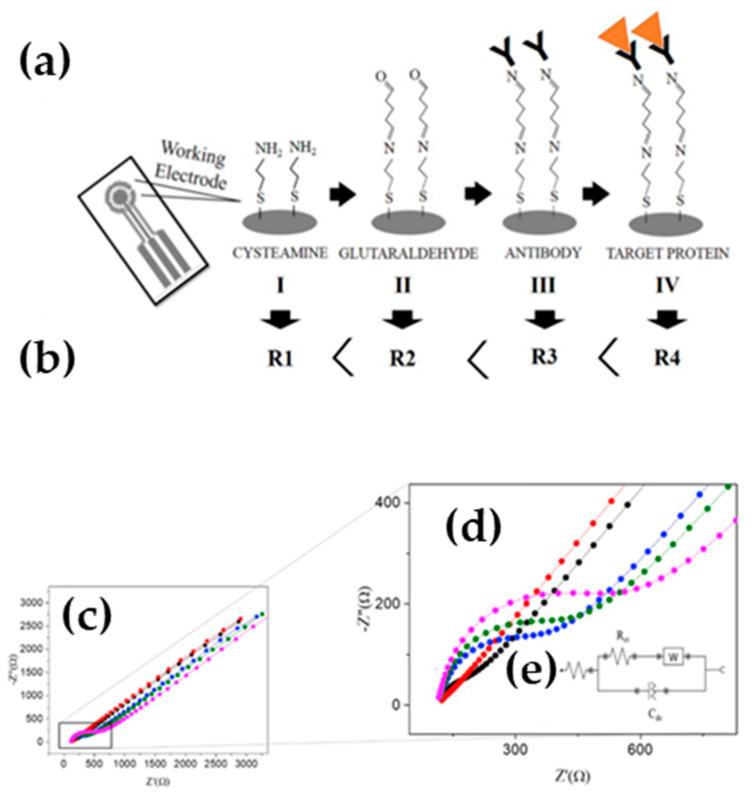
(**a**) Preparation scheme of the biosensor showing the steps of construction of the self-assembled monolayer, immobilization of the antibody and immunocomplex formation; (**b**) scheme showing increased resistance to electron transfer as further modifications are made on the working electrode surface; (**c**,**d**) Nyquist diagrams of (●) platinum electrode (Pt), (●) Pt-cysteamine, (●) Pt-cysteamine-glutaraldehyde, (●) Pt-cysteamine-glutaraldehyde-antibody and (●) Pt-cysteamine-glutaraldehyde-antibody-PARK7/DJ-1 protein; (**e**) equivalent circuit used for simulation of the experimental data, in the presence of redox couples. Reprinted with permission from reference [50]. Copyright © 2020 Elsevier B.V.

By means of square-wave voltammograms and EIS, the authors evaluated the electrochemical performance of the biosensor. The LOD obtained was 5.1 μmol L^−1^ and the sensitivity value was 0.016 μA mmol L^−1^. Figure 19a–b shows the preparation scheme of the biosensor and the increased resistance to electron transfer as further modifications were conducted. Second, EIS technique was used to evaluate the charge transfer resistance (R_ct_) response after each step of working electrode modification: (*i*) working electrode modification with cysteamine, (*ii*) binding of glutaraldehyde with cysteamine; (*iii*) antibodies immobilization by the interaction with glutaraldehyde and (*iv*) formation of the antigen- antibody complex. After the initial electrode modification with cysteamine, the system impedance decreased. This behavior is due to the protonation of the amine in solution, which provides the formation of a positively charged layer and consequent attraction of the electrochemical probe, causing a decrease in the impedance. On the other hand, with the presence of glutaraldehyde, the R_ct_ increases. In other words, the glutaraldehyde layer blocks the charge transfer between the solution and the surface of the flexible platinum electrode. As expected, when the antibodies are immobilized and interaction with the antigen (PARK7/DJ-1 protein) occurs, the system becomes more resistive.

## 4. Lab on a Chip (LOC), Electro-BIOsensors Fabricated with Common Plastics

In the last section of this review, a brief discussion about outstanding and complete electrochemical biosensors fabricated on flexible common plastics which are used as functional base substrates is reported. Flexible biosensors can be directly attached to the tissue surface and are designed in such a way as to withstand intense mechanical deformations, while maintaining stable performance. This class of sensors, also known as lab-on-a-chip (LOC) devices, is based on different materials employed as support, but here the attention is focused on plastic supports arranged in a single or layered configuration. Common thermoplastics as polyethylene (PE), polystyrene (PS), polyethylene terephthalate (PET), polypropylene (PP), polycarbonate (PC), thermoset (polyimide (PI, Kapton) are well employed for flexible biosensors because they can be easily turned into thin films and present low cost, inherent plasticity, hydrophobicity, good insulative properties, sufficient thermal stability, low coefficient of thermal expansion, structural resiliency against deformation and compatibility with fabrication processes.

### Wearable Biosensing Platforms

Among flexible biosensors, wearable devices are surely the ones which express the flexibility concept in the best way [139,140]. Herein, some examples of very innovative and challenging flexible biosensors composed by modified common thermoplastic and based on electrochemical detection methods is discussed. These electrochemical biosensors have been classified depending on the biologic biofluid analyzed. Sweat, saliva and tears are three main biofluids containing multiple physiologically relevant chemical constituents that be easily monitored in a continuous non-invasive real-time fashion [141,142,143].

**Figure 20:**
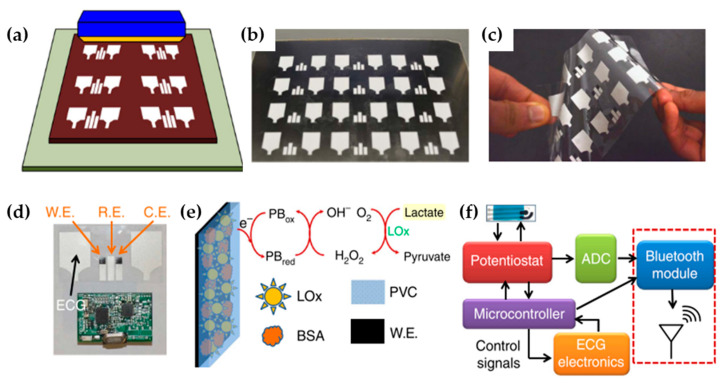
Fabrication and function of the Chem–Phys hybrid sensor patch. (**a**) Schematic showing the screen-printing process; (**b**) image of the Chem–Phys printing stencil; (**c**) An array of printed Chem–Phys flexible patches; (**d**) image of a Chem–Phys patch along with the wireless electronics; (**e**) Schematic showing the LOx-based lactate biosensor along with the enzymatic and detection reactions; (**f**) block diagram of the wireless readout circuit. Reprinted with permission from reference [144]. Copyright © Springer Nature Limited.

Wang’s group have fabricated a wearable device that can simultaneously measure biochemical (lactate) and electrophysiological parameters in the form of a single epidermal patch (Chem–Phys patch) and that comprises a screen-printed three-electrode amperometric lactate biosensor and two electrocardiogram electrodes [144]. The biosensors were fabricated via conventional low-cost screen- printing technique (Figure 20a–d) carried out on a 50-μm thin and highly flexible polyester substrate able to well adhere to the human skin. The working electrodes were first, printed using Prussian blue ink (high selective towards hydrogen peroxide, a byproduct of the enzymatic oxidation of lactate) and then functionalized and coated with a biocompatible biocatalytic layer (lactate oxidase (LOx)-modified Prussian blue). The reference electrode was printed using Ag/AgCl and, in order to avoid alternate electrically conductive pathway of the sweat, a printed hydrophobic layer of Ecoflex was used to separate the amperometric biosensor from the electrocardiogram electrodes.

The in vitro characterization of the lactate was carried out taking into account the typical range of concentration in sweat (0 to 25 mM) [145]. When the biosensor comes in contact with lactate, the immobilized LOx enzyme catalyzes the oxidation of lactate to generate pyruvate and H_2_O_2_. The Prussian blue transducer then selectively reduces the H_2_O_2_ to generate electrons to quantify the lactate concentration (Figure 20e). The biosensor responds linearly to the lactate concentrations in the desired range with a sensitivity of 96 nA/mM.

An impressive and advanced “smart wristband” or “smart headband” sensors designed for multiplexed in situ analysis using a wearable flexible integrated sensing array (FISA) enables real-time perspiration monitoring on the wrist and forehead during physical exercise was fabricated by Gao et al. [146]. Measurement of glucose and lactate was realized by chitosan entrapped glucose and lactate oxidase enzymes, respectively and amperometric measurements of the Prussian blue mediated reduction of the enzymatically produced hydrogen peroxide The sensor array incorporates the amperometric biosensors, a resistance-based temperature sensor and a flexible printed circuit board, all attached to a mechanically flexible polyethylene terephthalate (PET) substrate. The biosensor simultaneously and selectively measures sweat metabolites (such as glucose and lactate) and electrolytes (such as Na^+^ and K^+^), as well as the skin temperature (to calibrate the response of the sensors). The sensor array was fabricated using microengineering approaches as photolithography, electron beam evaporation, lift-off and oxygen plasma etching.

**Figure 21:**
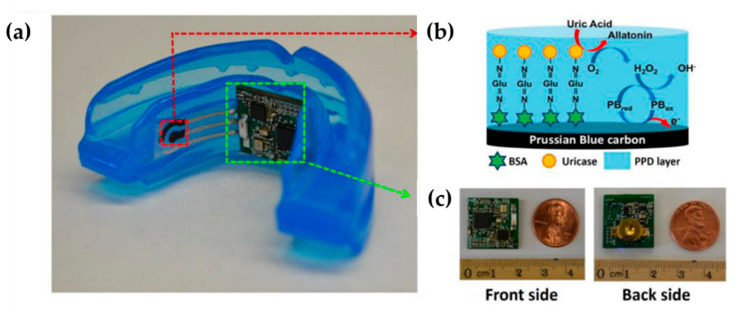
(**a**) Photograph of the mouthguard biosensor integrated with wireless amperometric circuit board; (**b**) reagent layer of the chemically modified printed Prussian blue carbon working electrode containing uricase for salivary uric acid (SUA) biosensor; (**c**) photograph of the wireless amperometric circuit board: front side (left) and back side (right). Reprinted with permission from reference [148]. Copyright © 2015 Elsevier B.V.

The first version of a mouthguard biosensor, a wearable salivary metabolite biosensor, was presented by Kim et al. [147] and it was based on the integration of a printable enzyme electrode. The authors monitored lactate by combining a lactate oxidase-modified biosensor and an amperometric detection of the enzymatically generated hydrogen peroxide Mouthguard biosensors were fabricated by screen-printing three separate layers on a flexible PET substrate. An Ag/AgCl conductive ink was printed first, to provide the reference electrode, second, the layer serving as the working and auxiliary electrodes was printed from a Prussian blue–graphite ink, and, finally, the third (insulator) layer, was printed by using the DuPont 5036 Dielectric paste. Subsequently, the printed electrode system was attached to the mouthguard body. The working electrode was subsequently modified in order to immobilize the enzyme (lactate oxidase (LOx)) by electropolymeric entrapment in a poly(o- phenylenediamine) (PPD) film. The last step consisted in the immersion of the mouthguard printable transducer in the polymerization solution to grow the LOx-entrapped PPD film. An improved version of this mouthguard biosensor was developed by the same group for the monitoring of salivary uric acid levels [148]. The enzyme (uricase)-modified screen printed electrode system was integrated onto a mouthguard platform along with anatomically miniaturized instrumentation electronics featuring a potentiostat, microcontroller and a Bluetooth low energy (BLE) transceiver enabling real time wireless transmission of the readings to remote electronic devices (Figure 21). The three separate layers on a flexible PET substrate were prepared using the same approach of the previous work [147].

**Figure 22:**
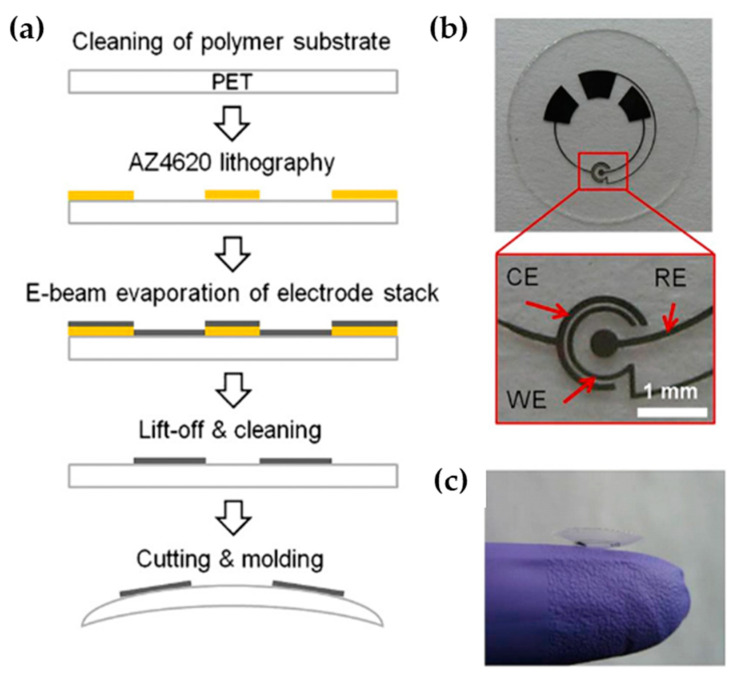
l-lactate sensor on contact lens. (**a**) Schematic of the fabrication process for sensors on the transparent polyethylene terephthalate (PET) substrate, which is molded into a contact lens shape; (**b**) flat substrate with sensing structure, interconnects and electrode pads for connection to the external potentiostat; (**c**) completed contact lens sensor held on a finger. Reprinted with permission from reference [150]. Copyright © 2011 Elsevier B.V.

New achievements on the non-invasive determination of glucose in tears by using suitably engineered contact lens biosensors has originated from Parviz’s team [149,150,151]. This group fabricated sensing devices based on flexible flat plastic supports, such as transparent PET thin films, shaped into a contact lens. At the point where irritation to the eye is minimal (that is, the periphery of a contact lens), PET surfaces were engineered to perform biosensing and sometimes wireless signal transduction, power supplying and transmittance of readings to a remote electronic device [151]. In a first attempt, a 3-electrode electrochemical cell along with three pads (used to make electrical connections between an external potentiostat and the sensor), were fabricated by evaporating Ti/Pd/Pt [149]. Glucose oxidase was immobilized on the working electrode using titania sol–gel film and Nafion was used to alleviate several potential interferences (ascorbic acid, lactate and urea). By using a similar fabrication process (Figure 22a) and glutaraldehyde cross-linked bovine serum albumin and lactate oxidase overcoated with medical grade polyurethane for biocompatibility, Thomas et al. demonstrated the monitoring of lactate in artificial tear fluid [150]. The device retained a stable current response for at least 24 h at room temperature, while an additional Nafion coating was applied for interference rejection. However, only a dual sensor configuration was found to be adequate for the rejection of ascorbic acid interference. The flat substrate with sensing structure, interconnects and electrode pads and the completed contact lens sensor is shown in Figure 22b,c. The same sensor-based contact lens biosensing concept was further elaborated for tear glucose monitoring with the addition of sensor read-out circuit, antenna and telecommunication circuit into a small chip [151].

## 5. Brief Conclusions and Future Outlooks

Herein, we wish to express our concluding thoughts and the outline future tasks for the different topics discussed in the present review, including various aspects related with the modification, application, sensor integration or the performance of polymers employed as electrochemical biosensors. By means of polymer modifications, new platforms for sensors suitable in biomedical application have emerged with a multitude of new and optimized properties. The electrochemical (amperometric, potentiometric,...) biosensors discussed in this review are fabricated by means of traditional and innovative modification techniques based on conducting polymers, as sensing material or based on inert polymers, modified to act as surface for the immobilization of the biosensing agent.

In the first part, a meticulous study about the different routes to optimize ICPs capacities for the electrochemical recognition of a wide range of biomolecules was performed. The collected information revealed that structural or superficial modifications with other materials, like metallic nanoparticles, graphene or its derivatives, improved the sensitivity of ICPs, achieving the ability to detect biomolecules with an important role in the diagnostics or treatments of diseases. Biomodifications also contribute to enhance the ICPs sensibility, in addition to the selectivity of the material toward specific bio-species, such as oncogenes or tumor marker. On the other hand, assemblies between ICPs and non-conducting polymers or biopolymers were employed to enhance other important qualities in a medical sensor, biocompatibility and hydrophilicity. These modifications are not limited to its individual use, in fact, several authors showed that more than one material or modification techniques could be merged to maximize the ICP behavior as an electro-biosensor. Based on the collected information new strategic modifications that combine the benefits of ICPs and conventional insulating polymers or biopolymers for their use in the biosensing field are required for efficient devices.

The second part of the review discusses "environmentally friendly" plastics, which represent a class of polymer in line with future trend of the scientific community as well of the polymer manufactures. The biocompatibility of polymer as chitosan can be suited to fabricate a new generation of biosensors exhibiting high electro-catalytic properties and it is also applicable for the estimation of biomolecules directly in human fluid samples. The synergistic effect of biopolymers and conducting materials, as graphene for example, can be achieved by means of traditional modification as drop casting or more advanced technique as molecular imprinting in the presence of ionic liquid. Another natural polymer of interest in biosensors is the polylactic acid (PLA), its biocompatibility was exploited for the immobilization of sensing agents, while, its biodegradability and good processability by 3D printing offer a suitable bioplatform for implantable sensing devices.

Eco/recycled plastics as LDPE, PET, PP are increasing utilized as support for the immobilization of enzymes and constitute powerful tools, even with a number of inherent disadvantages. Pioneer works based on LDPE modified with plasma technique or drop casting methodologies, previously discussed, proved that these modified plastics are a remarkable option for biosensors with good sensitivity and selectivity. They represent a new generation of biosensors, as demonstrated from the low number of scientific works published till now. There is often a trade-off between sensitivity and quick response and long-term stability and to achieve these goals an appropriate enzyme (or enzyme system) immobilization has to be investigated. Future trends may go in this direction. Taking into account that in such biosensors the plastic simultaneously act as support and mediator, the mechanical properties of the polymers as flexibility and resistance of the polymers are relevant and crucial to fabricate non-invasive sensors devices, as well the ease chemical modification of their surface to immobilize the biomolecules responsible of the sensing. The challenge is the fabrication of highly sensitive, stable, flexible and cheap biosensors, which may be employed in underdeveloped regions.

Finally, flexible devices based on recyclable plastics and characterized by a complex and complete electrochemical system were reported. In our opinion flexibility of the devices is an underrepresented aspect regarding structural biocompatibility that is fundamental in the process of insertion and that represent a challenge for scientist working with flexible devices.

## Figures and Tables

**Table 1:** Overview of some relevant publications on electrochemical detection based biosensors composed by polymers and on its type of modification, over the past 10 years.

Authors (Year)	Polymer Material ^1)^	Molecules Detected (EC Technique Used) ^2)^	Polymer Modified (Type of Modification) ^3)^	Limit of Detection ^4)^ (Detection Range)	Improvements
Poletti Papi et al. [32] (2017)	PPy	Glucose, bioproduct (CA)	PPy-AgNPs (Structural, reverse microemulsion method)	3.6 μM (25–2500 μmol L^−1^)	Sensitivity
Ansari et al. [33] (2019)	PAB	Glucose, bioproduct (CV)	PAB/AuNPs (Superficial, seed-assisted growth method)	0.4 µM (2–250 μM)	Sensitivity
Sha et al. [34] (2017)	PAni	Urea, bioproduct (CA)	PAni/Gr (Superficial, electrodeposition)	5.88 μM (10–200 mM)	Sensitivity
Weaver et al. [35] (2014)	PEDOT	Dopamine, neurotransmitters (CV)	PEDOT-GO (Structural, electropolymerization)	83 nM (1–40 mM)	Sensitivity and selectivity
Sun et al. [36] (2013)	PPy	Quercetin, flavonoid (DPV)	MIP PPy-Gr (Structural, MIT- electropolymerization)	4.8 × 10^−8^ mol/L (6.0 × 10^−4^–1.5 × 10^−2^ mM )	Sensitivity and selectivity
Radhakrishnan et al. [37] (2013)	PPy/PANi	DNA, biomolecule (DPV)	PPy/PANi/GA/ssDNA (Superficial, oxidative polymerization and biologic immobilization)	50 fM (10^−6^–10^−10^ mM)	Sensitivity and selectivity
Avelino et al. [38] (2016)	PAni	BCR/ABL (breakpoint cluster region- Abelson tyrosine kinase gene), oncogene (CV, EIS)	PAni-AuNPs/ssDNA (Structural and superficial, oxidative polymerization and biologic immobilization)	69.4 aM (10^−5^–10^−12^ mM)	Sensitivity and selectivity
Bayram and Akyilmaz [39] (2016)	PAni	Paracetamol, drug (CA)	PAni-cMWCNTs/ Bacillus sp./GA (Structural and superficial, electropolymerization and dip-coated)	2.9 μM (5–630 μM)	Sensitivity and selectivity
Molina et al. [40] (2018)	PPy	Serotonin, neurotransmitters (DPV)	PPy-*g*-PEG (Structural, “grafting through” technique)	0.07 µM (0.5–20 µM)	Biocompatibility
Cui et al. [41] (2016)	PEDOT	Alpha fetoprotein, tumor marker (EIS)	PEDOT-PEG/AuNPs (Structural and superficial, electropolymerization and biologic immobilization)	0.0003 fg/mL (0.001–10 fg/mL)	Hydrophilicity and selectivity
Devnani et al. [42] (2016)	CS	Noradrenaline (CV, SWV, EIS)	Graphene-chitosan (Structural, drop casting)	19.7 nM (200–1400 nM.)	Sensitivity and biocompatibility
Xia et al.[43] (2016)	CS	Bovine serum albumin (CV)	Chitosan/ionic liquid–graphene (Structural, molecular imprinting)	2 × 10^−11^ g/L (1.0 × 10^−10^–0 × 10^−4^ g/L)	Selectivity, sensitivity and biocompatibility
Ben-Yoav et al. [44] (2014)	CS	Clozapine (CV, SWV)	Catechol-modified chitosan (Structural, microfabrication technology)	0.1 μg/mL (0.1–10 μg/mL)	Sensitivity
Nordin et al. [45] (2016)	PLA	DNA (CV)	PLA-AuNPs (Superficial, Drop casting)	N.R.	Biocompatibility, mechanical properties,
Manzanares et al. [46] (2019)	PLA	Picric acid and Ascorbic acid (SWV)	Gr/PLA/proteinase K (Structural, Enzymatically sculptured 3D-printed electrode)	N.R.	Sensitivity
Das et al. [47] (2018)	PVA	Vitamin C, ascorbic acid (SWV)	Gr-iron oxide-polyvinyl alcohol (Structural)	0.234 μM (40–4100 μM)	Sensitivity and stability
Fabregat et al. [48] (2017)	PEDOT-, PNCPy, LDPE, PP, PCL, PS, LDPE	Dopamine (CV, CA)	PEDOT-, PNCPy, LDPE, PP, PCL, PS plasma treated (Superficial, cold plasma surface functionalization)	140 for PEDOT 750 nM for PNCPy (0.5–5 μM)	Sensitivity, selectivity, electric conductivity
Buendía et al. [49] (2017)	LDPE	Glucose (CA, CV)	LDPE-GOx (Superficial)	1.7 mM for PT-LDPE/GOx plasma treated 2 min (0–20 mM)	Sensitivity, selectivity, electric conductivity
Oliveira et al. [50] (2020)	Bio-PET sheets	Dopamine & Anti-PARK7/DJ-1 protein (SWV)	Pt electrode on Bio-PET (Superficial, microfabrication)	5.1 × 10^−3^ mM (3.5 ×10^−2^–8.0 × 10^−1^ mM)	Sensitivity and selectivity

^1)^ polypyrrole (PPy), poly(aniline blue) (PAB), polyaniline (PAni), poly(3,4-ethylenedioxythiophene) (PEDOT), polylactic acid, Chitosan(CS), (PLA), polyvinyl alcohol (PVA), poly(N-cyanoethylpyrrole) (PNCPy), low density polyethylene (LDPE), polypropylene (PP), polycarbonate (PC), polystyrene (PS) and polyethylene terephthalate (PET); ^2)^ EC technique (electrochemical technique used): cyclic voltammetry (CV), chronoamperometry (CA), differential pulse voltammetry (DPV), electrochemical impedance spectroscopy (EIS) and square wave voltammetry (SWV); ^3)^ Silver nanoparticles (AgNPs), gold nanoparticles (AuNPs), graphene (Gr), graphene oxide (GO), glutaraldehyde (GA), single strand deoxyribonucleic acid (ssDNA), carboxylated multiwalled carbon nanotubes (cMWCNTs), poly(ethylene glycol) (PEG) and glucose oxidase (GOx); ^4)^ Not reported (N.R.).
